# Anakinra Therapy for Non-cancer Inflammatory Diseases

**DOI:** 10.3389/fphar.2018.01157

**Published:** 2018-11-06

**Authors:** Giulio Cavalli, Charles A. Dinarello

**Affiliations:** ^1^Unit of Immunology, Rheumatology, Allergy and Rare Diseases, San Raffaele Hospital, Vita-Salute San Raffaele University, Milan, Italy; ^2^Department of Medicine, Radboud University Medical Center, Nijmegen, Netherlands; ^3^Department of Medicine, University of Colorado Denver, Denver, CO, United States

**Keywords:** interleukin 1, IL-1β, IL-1α, rheumatology, inflammation

## Abstract

Interleukin-1 (IL-1) is the prototypical inflammatory cytokine: two distinct ligands (IL-1α and IL-1β) bind the IL-1 type 1 receptor (IL-1R1) and induce a myriad of secondary inflammatory mediators, including prostaglandins, cytokines, and chemokines. IL-1α is constitutively present in endothelial and epithelial cells, whereas IL-1β is inducible in myeloid cells and released following cleavage by caspase-1. Over the past 30 years, IL-1-mediated inflammation has been established in a broad spectrum of diseases, ranging from rare autoinflammatory diseases to common conditions such as gout and rheumatoid arthritis (RA), type 2 diabetes, atherosclerosis, and acute myocardial infarction. Blocking IL-1 entered the clinical arena with anakinra, the recombinant form of the naturally occurring IL-1 receptor antagonist (IL-1Ra); IL-1Ra prevents the binding of IL-1α as well as IL-1β to IL-1R1. Quenching IL-1-mediated inflammation prevents the detrimental consequences of tissue damage and organ dysfunction. Although anakinra is presently approved for the treatment of RA and cryopyrin-associated periodic syndromes, off-label use of anakinra far exceeds its approved indications. Dosing of 100 mg of anakinra subcutaneously provides clinically evident benefits within days and for some diseases, anakinra has been used daily for over 12 years. Compared to other biologics, anakinra has an unparalleled record of safety: opportunistic infections, particularly *Mycobacterium tuberculosis*, are rare even in populations at risk for reactivation of latent infections. Because of this excellent safety profile and relative short duration of action, anakinra can also be used as a diagnostic tool for undefined diseases mediated by IL-1. Although anakinra is presently in clinical trials to treat cancer, this review focuses on anakinra treatment of acute as well as chronic inflammatory diseases.

## Introduction

### Historical Background of IL-1 and IL-1Ra

The history of interleukin 1 (IL-1) dates back to the purification of the endogenous fever-producing protein called leukocytic pyrogen, as reviewed in Dinarello (2015). During the purification of the leukocytic pyrogen, two fever-inducing proteins were observed with different molecular weights and distinct isoelectric points. Specifically, human blood monocytes produced both a high (35 kDa) as well as a low (15 kDa) molecular weight leukocytic pyrogen (Dinarello et al., 1974), with two distinct isoelectric points at 5.1 and 6.8, respectively (Dinarello et al., 1974). Murphy et al. (1981) also reported two leukocytic pyrogens, with isoelectric focusing points of 5.1 and 7.0 from rabbit cells. The specific biologic activity of purified human leukocytic pyrogen was first reported in 1977 as the induction of fever in rabbits at 10 ng/kg (Dinarello et al., 1977). Thus, the *in vivo* potency of IL-1 was established in 1977 and later confirmed in animals and humans with recombinant IL-1β. In 1979, based on the ability of purified human leukocytic pyrogen to enhance T-cell proliferation in response to antigen recognition, the name “leukocytic pyrogen,” or “lymphocyte activation factor” was replaced with the current nomenclature “IL-1” (Rosenwasser et al., 1979). The 1984 cDNA cloning of IL-1β in humans (Auron et al., 1984) and IL-1α in mice (Lomedico et al., 1984) univocally established that there were in fact two distinct genes coding for IL-1. Looking back today, the higher molecular weight fever-producing molecule was likely the IL-1α precursor, which unlike the IL-1β precursor is biologically active without processing. In contrast, the IL-1β precursor requires processing and proteolytic cleavage in order to generate the lower molecular weight and biologically active IL-1β.

Interleukin-1β exerts clinically marked pro-inflammatory effects at very low concentrations and correlations of circulating levels of IL-1β with disease severity is often not possible due to the limited sensitivity of immunoassays. Instead, human plasma has been assayed for IL-1 bioactivity by enhancement of PHA-induced proliferation of mouse thymocytes *in vitro*. This assay was reliable in that indirect readouts of IL-1 activity were found in plasma samples of subjects with endotoxemia and in women during the menstrual cycle (Cannon and Dinarello, 1985). The bioassay for IL-1 required chromatographic separation of each plasma sample in order to remove inhibitory proteins present in the plasma. Specifically, plasma from healthy human subjects was obtained before and after intravenous inoculum of a low dose of endotoxin. Before the administration of the endotoxin, the plasma fractions had no effect on thymocyte proliferation; however, 4 h after endotoxin administration, at the peak of the fever, fractions suppressed thymocyte proliferation. Thus, there was an endotoxin-inducible suppressor “factor” specifically inhibiting IL-1-mediated thymocyte proliferation in the circulation (Dinarello et al., 1981).

Subsequent to this observation, others reported the presence of a “specific” inhibitor of IL-1 bioactivity in supernatants of human monocytes (Arend et al., 1985) and in the serum and urine of children with systemic juvenile arthritis (Prieur et al., 1987). In 1987, this “IL-1 inhibitor” isolated from the urine was shown to prevent binding of IL-1 to cells (Seckinger et al., 1987), thus providing evidence for its mechanism of action. The IL-1 inhibitor was purified in 1990 (Hannum et al., 1990); the cDNA sequence first reported that same year (Eisenberg et al., 1990) and the term IL-1 receptor antagonist (IL-1Ra) was used for the first time in that report. Following the cDNA cloning of IL-1Ra, a radioimmunoassay for IL-1Ra was developed and used to assay the plasma samples from subjects during experimental endotoxemia. Endogenous IL-1Ra is found at very low levels in the circulation of healthy subjects (less than 200 rpg/mL), but levels steeply rise to a mean level of 1435 pg/mL 2 h after the infusion of endotoxin, and to top levels of 6400 pg/mL at the peak of the fever 4 h after infusion of endotoxin (Granowitz et al., 1991). Of note, IL-1β levels reached peak levels of only 20 pg/mL in the same samples. In that study, the kinetics of IL-1Ra matched the induction of the specific suppressor of IL-1-mediated thymocyte proliferation reported during experimental endotoxemia in 1981. In addition, the study also revealed that there was a molar excess of at least 100-fold IL-1Ra over IL-1β. It was not until the reports of subjects with a genetic deletion of IL-1Ra that the critical function of this endogenous inhibitor, which naturally suppresses IL-1 mediated inflammation, was fully revealed (Aksentijevich et al., 2009).

As stated in our review (Cavalli and Dinarello, 2018), in 1981 we described a circulating suppressor factor from humans during experimental endotoxemia as assayed for specific inhibition of IL-1 activity *in vitro* (Dinarello et al., 1981). We believe this circulating suppressor factor was the first description of IL-1Ra, and we confirmed our findings in a report published *in Lancet* in 1991 using a specific radioimmunoassay for IL-1Ra (Granowitz et al., 1991). However, in 1984, there was documentation from the group of Jean-Michel Dayer describing a specific inhibitor of IL-1 activity isolated from the urine of patients with monoblastic leukemia (Balavoine et al., 1984). This was an essential contribution to the history of the discovery of the antagonist. In 1985, there was another report from the Dayer laboratory “Collagenase- and PGE2-Stimulating Activity (Interleukin-1-Like) and Inhibitor in Urine from a Patient with Monocytic Leukemia,” as published in *Progress in Leukocyte Biology*, vol. 2 (New York, NY: Alan R. Liss, 1985 p. 429). These reports were followed by another publication in the *Journal of Clinical Investigation* (Balavoine et al., 1986). As stated in our Review, “the IL-1 inhibitor” isolated from the urine was shown to prevent binding of IL-1 to cells (Seckinger et al., 1987), thus providing for the first time evidence for its mechanism of action. Because of the widespread and beneficial use of anakinra (the recombinant form of the nature IL-1Ra) to treat human diseases, the contributions of Jean-Michel Dayer as well as those of William Arend are paramount.

### Synthesis and Release of IL-1β

**FIGURE 1:**
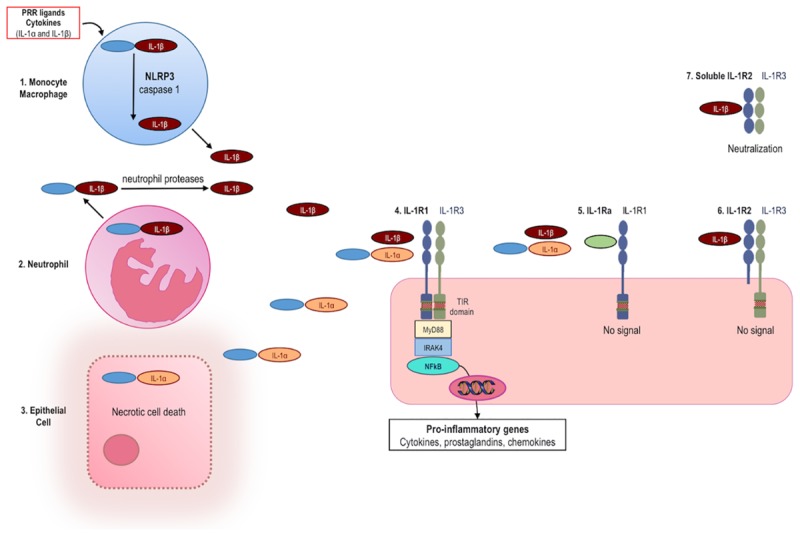
Production and release of IL-1, signaling and inhibition of IL-1 activities. (1) The IL-1β precursor is induced in monocytes/macrophages following engagement of pattern recognition receptors (PRR) or by pro-inflammatory cytokines, including IL-1α and IL-1β. IL-1β is synthesized as an inactive precursor (pro-IL-1β). Release of biologically active IL-1β takes place by enzymatic cleavage of the precursor protein by caspase-1. Activation of caspase-1 requires induction of the NLRP3 inflammasome. (2) Neutrophils release the IL-1β precursor into the extracellular space where it is cleaved to active IL-1β by neutrophil-derived proteases. (3) The IL-1α precursor is constitutively present in most epithelial cells and is fully active. Upon cell necrosis, the intracellular IL-1α precursor is released and acts as an alarmin. (4) Both IL-1α and IL-1β bind to IL-1 receptor type 1 (IL-1R1), which is followed by recruitment of the co-receptor IL-1R3 (formerly termed IL-1 receptor accessory protein, IL-1RAcP). The heterotrimer results in the approximation of the intracellular TIR domains of IL-1R1 and IL-1R3. MyD88, IL-1 receptor-associated kinase 4 (IRAK4), and NFκB are phosphorylated. NFκB induces transcription of pro-inflammatory genes. Mechanisms physiologically counteracting the activity of IL-1α and IL-1β include: (5) The IL-1 receptor antagonist (IL-1Ra, green) binds IL-1R1 and prevents binding of IL-1α and IL-1β, thereby resulting in no signal. (6) The IL-1 receptor type 2 (IL-1R2) preferentially binds IL-1SS, but lacking a cytoplasmic domain, acts as a decoy receptor and there is no signal. (7) Soluble IL-1R2 (extracellular domain only) binds IL-1β and forms a complex with soluble IL-1R3, resulting in neutralization of IL-1β.

Interleukin-1β is not produced or detectable with standard immunoassays in healthy tissues; rather, IL-1β is mainly produced by inflammatory cells of the myeloid compartment: blood monocytes, tissue macrophages, and dendritic cells. Figure 1 summarizes the mechanisms of IL-1 activation and signaling.

Production is stimulated by exogenous Toll-like receptor (TLR) agonists or by endogenous cytokines such as TNFα (Dinarello et al., 1987). IL-1α and IL-1β induce themselves. This self-sustained induction of IL-1 is a key mechanism of autoinflammation. In order to prevent unwanted release and runaway inflammation, IL-1β is synthesized as an inactive precursor, whose activation is contingent on proteolytic cleavage by caspase-1, an intracellular cysteine protease. In turn, activation of caspase-1 requires the oligomerization and assembly of the “inflammasome,” a complex of intracellular proteins (Martinon et al., 2009). Once activated, caspase-1 cleaves the N-terminal amino acid of the inactive IL-1β precursor, thus enabling the release of the processed, biologically active form of this cytokine. Assembly and activation of the inflammasome thereby represents a critical safety mechanism preventing deregulated release of IL-1β. Unrestricted activation of caspase-1 and secretion of IL-1β lead to systemic and multi-organ sterile inflammation, which characterizes autoinflammatory diseases (Hoffman and Wanderer, 2011).

### Anakinra Reveals the Nature of Autoinflammatory Disorders

The term IL-1 is often used without distinguishing between the two gene products, IL-1α and IL-1β. This is because both cytokines bind to the same signaling receptor, the IL-1 receptor 1 (IL-1R1), and hence there is no significant difference between the biological activities of either cytokine. IL-1Ra, also an endogenous member of the IL-1 family, binds to the IL-1R1 and therefore blocks the activity of both IL-1α and IL-1β. A recombinant form of IL-1Ra (anakinra) is used to treat a broad variety of diseases, ranging from common conditions such as rheumatoid arthritis (RA), gout, and idiopathic pericarditis, to rare hereditary diseases. Specific mutations in diseases such as familial Mediterranean fever (FMF) and cryopyrin-associated periodic syndrome (CAPS) result in deregulated release of active IL-1β, which is clinically manifested as periodic fevers with systemic and local inflammation. These diseases do not involve T-lymphocytes, which characteristically represent the effector cells of every autoimmune disease, nor autoantibodies. Therefore, these diseases are not considered autoimmune diseases, but rather termed “autoinflammatory” syndromes. In autoinflammatory diseases, the effector cell is a myeloid cell, characteristically a monocyte or macrophage (Dinarello et al., 2012). The central role of IL-1 in the pathogenesis of autoinflammation is well established. Monocytes from patients with autoinflammatory diseases release more IL-1β, but not TNFα, compared to healthy persons (Pascual et al., 2005; Goldbach-Mansky et al., 2006; Gattorno et al., 2007).

**FIGURE 2:**
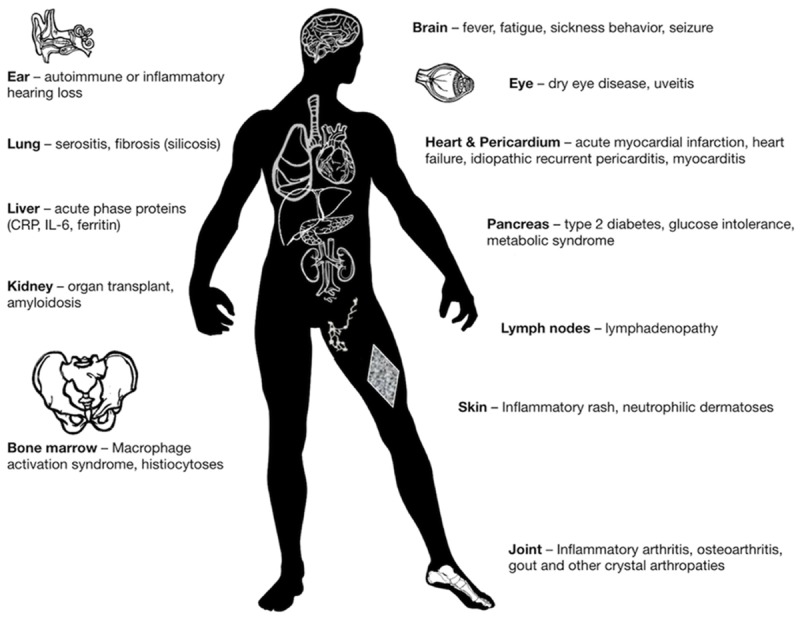
Clinical manifestations of IL-1-mediated inflammation, which are reversible upon treatment with anakinra.

Autoinflammatory diseases can be regarded as a “natural experiment”, which reveals the clinical and pathologic consequences of deregulated IL-1-mediated inflammation in humans. Lessons from autoinflammatory diseases extend and apply beyond this group of rare conditions: deregulated activation of the myeloid compartment and IL-1 also mediate several common diseases, which can also be classified as autoinflammatory disorders (i.e., gout, pericarditis), or at least include autoinflammation as part of disease pathogenesis (i.e., heart failure, diabetes, myocarditis; Cavalli et al., 2016a; Hayashi et al., 2016; Netea et al., 2017). Because of the safety and rapid onset of action, IL-1 inhibition with anakinra can be used as a diagnostic as well as a treatment tool for patients with undefined signs or symptoms of autoinflammation (Harrison et al., 2016). Figure 2 illustrates the broad variety of organ manifestations of IL-1-mediated inflammation, which can be treated with anakinra.

## Autoinflammatory Diseases

Autoinflammatory diseases are a spectrum of hereditary or multifactorial conditions variably manifested with clinical and hematologic features of IL-1-mediated inflammation: these include fever, fatigue, myalgia, arthralgia, arthritis, serositis, gastrointestinal involvement, skin rashes, and multi-organ involvement, often accompanied by neutrophilia and elevated inflammatory markers. Most autoinflammatory diseases occur in recurrent flares. In some autoinflammatory diseases, causative mutations resulting in deregulated release of active IL-1 have been identified. However, in other autoinflammatory diseases, a specific mutation to account for excessive IL-1 activity has not yet been determined; in some cases, defects in regulatory molecules counteracting the biologic activity of IL-1 can be determined, or postulated (Aksentijevich et al., 2009; Cavalli et al., 2016b; Cavalli et al., 2017c,d; Ballak et al., 2018; Cavalli and Dinarello, 2018). Regardless of the underlying mechanisms, disease manifestations are characteristically controlled by IL-1 blockade with anakinra; as different pharmacokinetics result in more prolonged duration of action, neutralizing antibodies directed against IL-1β (canakinumab) represent an alternative in patients enduring frequent disease flares. The efficacy of anakinra in the treatment of these conditions is discussed below; more detailed lists of hereditary as well as non-hereditary inflammatory diseases responsive to anakinra treatment are provided in Tables 1, 2.

**Table 1:** Anakinra for hereditary systemic inflammatory diseases.

Familial Mediterranean fever (FMF; Meinzer et al., 2011; Ozen et al., 2011)
CAPS (Hawkins et al., 2003; Goldbach-Mansky et al., 2006; Kullenberg et al., 2016)
TRAPS (Simon et al., 2004; Gattorno et al., 2008a)
PAPA (Dierselhuis et al., 2005; Brenner et al., 2009; Braun-Falco et al., 2011; Schellevis et al., 2011)
PASH (Braun-Falco et al., 2011; Marzano et al., 2013)
DIRA (Aksentijevich et al., 2009; Reddy et al., 2009; Sakran et al., 2013)
Blau syndrome/granulomatous arthritis (Arostegui et al., 2007; Punzi et al., 2011)
Mevalonate kinase deficiency/hyper-IgD syndrome (Ruiz Gomez et al., 2012)
Majeed syndrome (Herlin et al., 2013)
NLRP12 autoinflammatory syndrome (Jeru, 2011)

**Table 2:** Anakinra for systemic and local inflammatory diseases.

Schnitzler syndrome (Ryan et al., 2008)
Behçet disease (Cantarini et al., 2015b)
Secondary amyloidosis (Moser et al., 2009; Ait-Abdesselam et al., 2011; Stankovic Stojanovic et al., 2012)
Henoch–Schonlein purpura (Boyer et al., 2011)
Idiopathic recurrent pericarditis (Picco et al., 2009; Brucato et al., 2016)
Systemic-onset juvenile idiopathic arthritis (Gattorno et al., 2008b; Vastert et al., 2014)
Adult-onset still disease (Fitzgerald et al., 2005; Cavalli et al., 2015b; Colafrancesco et al., 2017)
Macrophage activation syndrome (Gattorno et al., 2008b; Miettunen et al., 2012; Rajasekaran et al., 2014; Vastert et al., 2014; Sonmez et al., 2018)
Sweet’s syndrome/neutrophilic dermatoses (Delluc et al., 2008; Kluger et al., 2011; Pazyar et al., 2012; Belani et al., 2013)
Neutrophilic panniculitis (Behrens et al., 2006; Aronson and Worobec, 2010; Lipsker et al., 2010)
Erdheim–Chester/histiocytoses (Aouba et al., 2010; Diamond et al., 2016; Tomelleri et al., 2018)
SAPHO (Colina et al., 2010; Eleftheriou et al., 2011; Wendling et al., 2012)
PFAPA (Stojanov et al., 2011; Cantarini et al., 2012a)
Multicentric Castleman disease (Galeotti et al., 2008)
Jessner–Kanof disease (Sparsa et al., 2012)
Primary Sjögren syndrome fatigue (Norheim et al., 2012)
Kawasaki disease (Cohen et al., 2012)
Colitis in chronic granulomatous disease (van de Veerdonk et al., 2011)
Hidradenitis suppurativa (Tzanetakou et al., 2016)
Autoimmune inner ear disease (Vambutas et al., 2014)

### Cryopyrin-Associated Periodic Syndromes (CAPS)

The term “CAPS” encompasses a spectrum of three hereditary diseases: familial cold autoinflammatory syndrome (FCAS), Muckle–Wells syndrome (MWS), and neonatal onset multi-inflammatory diseases (NOMID). The clinical phenotypes of FCAS, MWS, and NOMID are characterized by escalating severity, ranging from self-limited episodes of fever, myalgia, and skin rash (FCAS), to chronic systemic and organ-specific inflammation with major complications (NOMID). The pathologic hallmark of CAPS is the presence of activating mutations in the NLRP3 inflammasome, which result in induction of caspase 1 and deregulated release of IL-1β (Agostini et al., 2004; Goldbach-Mansky et al., 2006; Gattorno et al., 2007). Anakinra, as well as other IL-1-blocking agents, is dramatically effective in the treatment of CAPS (reviewed in Cavalli and Dinarello, 2015).

### Familial Mediterranean Fever (FMF) and Amyloidosis

Familial Mediterranean fever is a prototypical autoinflammatory disease characterized by recurrent bouts of fever, rash, and serositis, which are typically self-limited in 3–5 days. The disease is autosomal recessive, and determined by mutations in the MEFV gene, encoding for pyrin, a protein involved in inflammasome formation, activation of caspase 1, and release of active IL-1β (Chae et al., 2006). Colchicine is the cornerstone of treatment for FMF, but is ineffective in some patients. Patients refractory to conventional treatment are successfully treated with anakinra (Meinzer et al., 2011; Ozen et al., 2011).

Interleukin-1 induces serum amyloid A (SAA), which is thereby commonly elevated in several chronic inflammatory diseases. Progressive deposition of amyloid fibrils in tissues results in amyloidosis, a condition leading to severe organ dysfunction, including lethal kidney or heart failure, which are major causes of death in untreated FMF and CAPS. However, effective dampening of chronic inflammation with IL-1 blockers prevents progression to amyloidosis and organ failure (Moser et al., 2009; Ait-Abdesselam et al., 2011; Ozen et al., 2011; Stankovic Stojanovic et al., 2012). Intriguingly, amyloid deposition leading to organ dysfunction is also a feature of other, multifactorial conditions in which IL-1 has emerged as a pivotal pathologic mediator: amyloidosis of insulin-producing islets and brain characterizes T2D and Alzheimer disease, respectively (Tan et al., 2007; Masters et al., 2010). In both conditions, IL-1 blockade may represent a suitable therapeutic strategy to hinder disease progression.

### TNF-Receptor Associated Periodic Syndrome (TRAPS)

TNF-receptor associated periodic syndrome (TRAPS) is an autosomal dominant disease caused by mutations in TNF-receptor type 1 (McDermott et al., 1999), and clinically manifested as recurrent flares of fever, rash, and serositis. A deficiency of soluble TNFα receptors, which neutralizes circulating TNFα, was postulated. Nevertheless, clinical response to TNFα inhibition is only partial, and even absent in many patients. Current understanding rather postulates a deficit in membrane translocation of TNF-receptor type 1, which leads to an unfolded protein response, cellular stress, and consequent release of IL-1β (Cantarini et al., 2012b). This view is also substantiated by clinical efficacy of anakinra, even in refractory cases (Simon et al., 2004; Gattorno et al., 2008a).

### Hyper-IgD Syndrome (HIDS)

Also known as mevalonate kinase deficiency, Hyper-IgD syndrome (HIDS) is an autosomal recessive autoinflammatory disorder characterized by recurrent fever, myalgia, skin rash, and lymphadenopathy. Episodes usually last 4–6 days and can be triggered by infections. Multiple intracellular pathways link mevalonate kinase deficiency with deregulated release of IL-1 production (Stoffels and Simon, 2011). Consistently, IL-1 inhibitors effectively reduce the frequency and severity of the attacks of HIDS (Bodar et al., 2011).

### Adult Onset Still’s Disease (AOSD) and Systemic Onset Juvenile Idiopathic Arthritis (SOJIA)

Adult onset Still’s disease (AOSD) is a rare, systemic inflammatory syndrome characterized by arthritis, fever, rash, multi-organ inflammation, and strikingly elevated serum inflammatory indexes, particularly ferritin. Consistent with observations that the NLRP3 inflammasome is highly expressed and activated in AOSD (Hsieh et al., 2017), IL-1β blockade, even with anakinra as monotherapy, represents the mainstay of biologic treatment and effectively controls disease manifestations (Fitzgerald et al., 2005; Naumann et al., 2010; Cavalli et al., 2015b; Colafrancesco et al., 2017; Junge et al., 2017).

Rather than being distinct clinical entities, SOJIA and AOSD are considered different manifestations of the same disease, occurring in infancy and adulthood, respectively. The efficacy of anakinra in SOJIA is thereby not unexpected, even in patients refractory to treatment with steroids, methotrexate, or TNFα blockers (Quartier et al., 2011). Both in AOSD and SOJIA, two distinct clinical phenotypes can be identified. One is characterized by rampant systemic inflammation with neutrophilia and elevated acute phase proteins, and by a lower number of inflamed joints: this form is dramatically and characteristically responsive to IL-1 blockade (Gattorno et al., 2008b). On the other hand, a clinical phenotype characterized by more severe arthritis and limited systemic inflammation may not respond as brilliantly to IL-1 inhibition.

Of note, treatment of SOJIA poses challenges beyond the mere achievement of disease control, related to the problematic use of immunosuppressive therapies in a pediatric population. For instance, steroid treatment is associated with growth retardation. In this scenario, IL-1 inhibition may be particularly advantageous, as treatment with anakinra or canakinumab results in reduced glucocorticoid dosing and catch-up growth (Ruperto et al., 2012). A prospective study evaluated anakinra (2 mg/kg) as a first-line drug in 20 children with new-onset SOJIA, and documented a near complete clinical response within 3 months of treatment initiation, which was sustained at 32 months of follow-up and allowed most patients to discontinue treatment (Vastert et al., 2014).

### Schnitzler Syndrome (SchS)

Schnitzler syndrome (SchS) is characterized by chronic urticarial, fever, and development of hematopoietic malignancies, particularly Waldenström macroglobulinemia. The SchS International Registry reports nearly 100% efficacy with anakinra treatment, which leads to clinical improvement within hours and remission within days (Ryan et al., 2008). Remission upon treatment is durable, but disease flares may occur at discontinuation (Mertens and Singh, 2009a). The efficacy of anakinra is SchS is so distinctive that diagnosis should be reconsidered in the event of treatment failure. Canakinumab is also highly effective in SchS (de Koning et al., 2013). Of note, a recent study reported that some patients with SchS have myeloid-lineage restricted somatic mosaicism for mutations in NLRP3, which is associated with increased IL-1 activity in monocytes. This phenomenon likely explains the late onset of disease in some patients (de Koning et al., 2015).

## Anakinra for the Heart

The central role of IL-1-mediated inflammation is established in the pathogenesis of atherosclerosis, ischemia-reperfusion injury, cardiac remodeling, and myocardial infarction (Pomerantz et al., 2001; Kamari et al., 2007; Duewell et al., 2010; Ridker et al., 2017). The beneficial effects of IL-1 blockade with anakinra in heart disease are discussed hereby, and summarized in Table 3.

**Table 3:** Anakinra for the heart.

↓ Inflammation in acute myocardial infarction
↑ Exercise performance in heart failure
↑ Oxygen consumption in heart failure
↓ Systemic inflammation in heart failure
↓ Hospitalizations for recurrent acute heart failure
↓ Pain and inflammation in recurrent idiopathic pericarditis
↑ Function in acute myocarditis and heart failure
↑ Exercise tolerance in heart failure associated with rheumatoid arthritis

### Atherosclerosis

Chronic inflammation is central to the pathogenesis of atherosclerosis (Libby et al., 2002), and IL-1 specifically promotes the formation, growth, and rupture of vascular atherosclerotic plaques, which account for ischemic cardiovascular complications (Peiro et al., 2017; Buckley and Abbate, 2018). Both IL-1β and IL-1α are highly expressed in atherosclerotic lesions, and promote recruitment of leukocytes by inducing endothelial cells to express adhesion molecules; in addition, IL-1 impairs vasodilation while inducing oxidative stress and pro-coagulant mediators (Peiro et al., 2017). Experimental pre-clinical evidence shows that many of these effects can be reversed by IL-1β inhibition, thus pointing at selective pharmacological blockade as a suitable treatment strategy to dampen progression of atherosclerotic lesions (Peiro et al., 2017). Although the ability of anakinra or other IL-1β blocking drugs to prevent progression of atherosclerosis in humans has not been specifically studied, IL-1 inhibition with anakinra and canakinumab proved beneficial in the treatment of major clinical complications of atherosclerosis, such as acute myocardial infarction and ischemic cardiovascular disease, as detailed in the following sections of this review.

### Acute Myocardial Infarction

The first studies of anakinra in acute ischemic heart disease involved patients who had suffered a ST-elevated myocardial infarction (STEMI; Abbate et al., 2010, 2013). In these studies, anakinra 100 mg daily was administered subcutaneously for 2 weeks, following stent placement and in addition to optimal standard of care. Seventy-two hours after the acute event, despite optimal standard of care, inflammation develops due to myocardial ischemia; CRP reaches peak levels, which correlate with the size of the infarcted area. Anakinra treatment resulted in a significant reduction in CRP levels (Abbate et al., 2013), thus reducing the progressive inflammatory response and myocardial damage. Infiltration of neutrophils and monocytes into the area surrounding the ischemic tissue contributes to further damage, which is significantly reduced by anakinra treatment in animal studies (Toldo et al., 2013). Twelve weeks following myocardial infarction, heart function of patients was evaluated as residual left ventricular ejection fraction. Compared to the placebo-treated group, anakinra-treated patients exhibited improved functional status, but did not reach statistical significance (Abbate et al., 2013). A second trial was performed with 30 patients (Abbate et al., 2013). Again, anakinra significantly reduced CRP levels 72 h after myocardial infarction; after 10–14 weeks, this reduction in CRP correlated with a reduction in left ventricular end-systolic volume (Abbate et al., 2013). Patients treated with anakinra exhibited an overall reduction in the development of heart failure (New York Heart Association Grade III and IV) compared to placebo-treated patients (Abbate et al., 2013).

Subsequent studies confirmed that anakinra treatment effectively dampens inflammation associated with myocardial infarction. Anakinra treatment was started after standard of care for the acute event and protracted for 14 days in 182 patients with non-STEMI myocardial infarction (Morton et al., 2015). For 7 days following the acute event, a significant reduction in CPR was observed in patients receiving anakinra compared to placebo (21 compared to 43 mg day/L); levels rose again 16 days after cessation of anakinra (Morton et al., 2015).

In the massive Canakinumab Anti-inflammatory Thrombosis Outcomes Study (CANTOS), 10,061 patients with prior myocardial infarction and evidence of systemic inflammation as determined by elevated serum CRP were randomized to receive either placebo or canakinumab (50, 150, or 300 mg every 3 months). At 48 months, patients treated with canakinumab 150 mg exhibited a 15% reduction in the primary endpoint of recurrent non-fatal myocardial infarction and non-fatal stroke, or cardiovascular death, as well as reduced need for coronary revascularization, compared to placebo (Ridker et al., 2017). These definitive results indicate that IL-1β blockade with canakinumab in patients with atherosclerotic cardiovascular disease can prevent recurrent cardiovascular events. Of note, this benefit was closely related to suppression of inflammation, as patients exhibiting the greatest reduction in CRP had improved survival upon treatment (Ridker et al., 2018).

### Heart Failure

Several years ago, *ex vivo* studies with human atrial heart strips revealed that IL-1β suppresses cardiac contractility, even at picomolar concentrations (Cain et al., 1999). In recent years, various studies examined the effects of anakinra on heart failure with poor exercise tolerance and signs of systemic inflammation. For example, mice treated with a single dose of recombinant human IL-1β have a 76% reduction in response to isoproterenol and a 32% reduction in left ventricular function. In a clinical trial, seven patients with heart failure and markers of systemic inflammation despite standard of care treatment received 100 mg of anakinra daily for 14 days. Compared to baseline, treatment with anakinra was associated with a statistically significant improvement in oxygen consumption, a marker of exercise capability (Van Tassell et al., 2012). This study first established a role for anakinra treatment in patients with refractory heart failure.

Besides impaired left ventricular contractility, heart failure with preserved ejection fraction can also occur and be associated with reduced exercise tolerance. When patients with this condition were treated with anakinra 100 mg daily for 14 days in a double-blind, randomized, placebo controlled study, patients receiving treatment exhibited a significant increase in oxygen consumption of 1.2 mL/kg/min and a concomitant 74% reduction in CRP.

Patients with acute, decompensated heart failure often exhibit signs of systemic inflammation. Thirty patients with acute decompensated heart failure, ejection fraction less than 40%, and elevated CRP were randomized to receive either anakinra or placebo (Van Tassell et al., 2016). Upon entering the trial, patients received either 100 mg anakinra or placebo twice daily for 3 days followed by 11 days of once daily dosing. Three days into the trial, CRP decreased by 61% in the anakinra group compared to the placebo-treated group (Van Tassell et al., 2016). Although the study was not powered to determine a clinical benefit, it showed that IL-1β inhibition with IL-1 receptor blockade reduced the systemic inflammation associated with acute heart failure.

In all these trials on heart failure, patients received anakinra for only 14 days. Although clinical and objective data indicate a functional improvement as well as reduced inflammation already with short-term treatment, it is likely that a prolonged course of anakinra would result in a more marked benefit. For example, patients hospitalized with an episode of acute decompensated heart failure are at high risk for repeated hospitalizations due to recurrent episodes. Therefore, a trial was conducted comparing two different treatment durations (2 versus 12 weeks) of anakinra 100 mg daily in patients discharged from the hospital following an episode of acute decompensated heart failure. In this study, patients treated with 12 weeks of anakinra had reduced hospital readmission rates and improved aerobic capacity, oxygen consumption, and quality of life compared to patients receiving either placebo or 2 weeks of anakinra (Van Tassell et al., 2017). Of note, patients receiving anakinra for RA exhibited improved cardiac contractility, even within 3 h of a single administration (Ikonomidis et al., 2008).

### Idiopathic Recurrent Pericarditis

Pericarditis is an autoinflammatory manifestation, often encountered as part of the clinical spectrum of inherited autoinflammatory disorders such as TRAPS, FMF, and CAPS, and successfully treated with anakinra (Cantarini et al., 2010; Kuemmerle-Deschner et al., 2011b). Patients with AOSD also can have bouts of pericarditis (Gerfaud-Valentin et al., 2014) and respond to anakinra (Cavalli et al., 2015b; Colafrancesco et al., 2017).

However, inflammation of pericardium can occur as an isolated, recurrent manifestation with no clear genetic predisposition (idiopathic recurrent pericarditis), which often develops after a viral illness. Anakinra is highly effective in treating these patients and provides a rapid and sustained reduction in pain, particularly in patients refractory to conventional treatment with colchicine (Scott et al., 2011; Imazio, 2014; Brucato et al., 2016). Treating pericarditis with etanercept or infliximab has not been successful (Ambrose and O’Connell, 2007; Devasahayam et al., 2012).

### Myocarditis and Dilated Cardiomyopathy

Clinical observations indicate a central role of IL-1 in the pathogenesis of cardiac inflammation. For example, myocardial involvement is part of the clinical spectrum of inflammatory (Cavalli et al., 2013b, 2014; Campochiaro et al., 2015) or autoinflammatory diseases (Lopetuso et al., 2013; van de Veerdonk and Netea, 2013), such as AOSD or SOJIA, which are characteristically mediated by IL-1. IL-1 blockade is highly effective in these conditions (Cavalli and Dinarello, 2015), as exemplified by several published cases of myocarditis associated with SoJIA and AOSD and promptly controlled by anakinra (Raffeiner et al., 2011; Movva et al., 2013; Choi et al., 2014; Cavalli et al., 2015b). However, there is emerging evidence that anakinra can be effective in the treatment of fulminant myocarditis irrespective of the initiating trigger or underlying condition (Raffeiner et al., 2011; Cavalli et al., 2016c, 2017b; De Luca et al., 2018b). In patients with myocarditis-associated acute heart failure, beneficial effects of anakinra on myocardial contractile function are particularly striking, and generally consistent with the observed benefit in patients with heart failure. It remains to be determined whether anakinra would increase myocardial function in non-acute myocarditis as it does in the acute condition. Blocking TNFα in myocarditis is contraindicated. There are case reports of anakinra treatment for myocarditis in patients non-responsive to anti-IL-6 (Waghmare et al., 2015).

A recent report described the efficacy of anakinra in a patient with dilated cardiomyopathy, a severe, irreversible heart disease characterized by left ventricular systolic dysfunction and dilation, which are not explained by abnormal loading or coronary artery disease. It is likely that several etiologic types of myocardial damage confluence in this common end-stage condition, which is histologically characterized by loss of contractile tissue, remodeling, and fibrosis. In the patient described in this study, histologic analyses of heart specimens had revealed subtle inflammation: treatment with IL-1 inhibition was thereby started, and led to a prompt clinical improvement in contractile function and arrhythmic burden (De Luca et al., 2018a). These results point at a possible role of low-grade chronic inflammation in the pathogenesis of dilated cardiomyopathy.

## Diabetes and Metabolic Syndrome

Interleukin-1-mediated inflammation plays a critical role in the progressive loss of β cells, which characterizes progression from insulin resistance to T2D (van Asseldonk et al., 2011). Specifically, IL-1β gene expression is dramatically elevated in β-cells of T2D patients compared to controls (Donath and Shoelson, 2011), whereas IL-1Ra levels are locally reduced and insufficient to protect β-cells from inflammation-mediated damage (Boni-Schnetzler et al., 2018).

Mechanistically, high glucose concentrations trigger β cells to produce IL-1β (Maedler et al., 2002), which in turn contributes to β-cell loss by promoting deposition of amyloid (Masters et al., 2010). These pivotal observations delineated a new concept of T2D as a chronic inflammatory disease, in which IL-1-driven inflammation results in progressive loss of β cell function (Donath and Shoelson, 2011), and provided rationale for testing anakinra in T2D. In a randomized trial, treatment with anakinra for 13 weeks led to improved insulin production and glycemic control. Reduction in IL-1-mediated inflammation was confirmed by decreased levels of CRP and IL-6 (Larsen et al., 2007), and likely particularly relevant and sustained at the islet level, as treatment responders required 66% less insulin to maintain glycemic control in the 39 weeks following discontinuation (Larsen et al., 2009). This pilot study led to subsequent, large trials of anti-IL-1β monoclonal antibodies gevokizumab, LY2189102, and – particularly – canakinumab in T2D, which all confirmed clinical benefits (Cavelti-Weder et al., 2012; Rissanen et al., 2012; Sloan-Lancaster et al., 2013).

Human fat tissue is an inflammatory environment in which infiltrating macrophages produce IL-1β (Stienstra et al., 2011). Anakinra treatment was thereby also evaluated in non-diabetic patients with metabolic syndrome, and was associated with a decrease in CRP and a corresponding increase in disposition index, thus reflecting improved β cell function (van Asseldonk et al., 2011).

Given the association between cardiovascular disease and T2D, the potential of IL-1 blocking agents to improve cardiovascular health and glucose metabolism was assessed in the large CANTOS trial, which determined that treatment with the anti-IL-1β monoclonal antibody canakinumab reduces re-occurrence of ischemic events in patients with prior cardiovascular accidents (Ridker et al., 2017). The CANTOS trial met its primary and secondary endpoints in both T2D subjects as well as in those without diabetes. Consistent with anakinra treatment in T2D, canakinumab reduced HbA1c during the first 6–9 months of treatment (Everett et al., 2018). However, after nearly 4 years of canakinumab treatment, prevention of progression to overt T2D in subjects with impaired glucose tolerance at enrollment was not observed and there was no sustained improvement in glycemic control (Everett et al., 2018). Thus, there is likely a role for IL-1α in T2D. Indeed, two studies in recent onset T1D tested anakinra versus canakinumab. Increased C-peptide was reported in subjects treated with anakinra but not canakinumab (Moran et al., 2013).

## Joint Diseases

### Rheumatoid Arthritis and Associated Comorbidities

The efficacy of anakinra treatment in RA was evaluated in several controlled studies (Mertens and Singh, 2009b). Anakinra monotherapy or in association with methotrexate significantly reduced disease severity, joint space narrowing, radiographic joint damage, and bone erosions, while also improving quality of life (Bresnihan et al., 2004). However, other biologics, including TNFα blockers, dominate the field of biologic treatments for RA. No direct comparison is available between the efficacy of IL-1 blockade and the overwhelming number of competing biologic agents; based upon indirect comparisons, anakinra seems moderately efficacious (Singh et al., 2010). Currently, anakinra is mostly administered to those RA patients in whom other biologics proved ineffective or are contraindicated, for example, due to previous malignancy or recurrent infections. In patients refractory to anti-TNFα therapy, anakinra was shown to be effective in controlling disease activity (Genant et al., 2001; Bresnihan et al., 2004; Botsios et al., 2007). Similar to anakinra, the anti-IL-1β monoclonal antibody canakinumab has reduced disease severity in RA patients, including those unresponsive to anti-TNFα therapies (Alten et al., 2011); however, unlike anakinra, long-term preservation of joint function with canakinumab remains unstudied.

Compared to the general population, RA patients exhibit a higher incidence of T2D and cardiovascular events (Primdahl et al., 2013). In particular, infection and cardiovascular disease are the leading causes of death in RA patients, whereas T2D and metabolic syndrome are burdensome comorbidities (Kelly and Hamilton, 2007). An ideal treatment should thereby not only reduce pain and prevent articular damage, but also aim at treating associated comorbidities with minimal adverse effects. Given the IL-1-mediated nature of these comorbidities, and the documented favorable effects of IL-1 blockade on cardiovascular and metabolic diseases (discussed above in the present review; Dinarello et al., 2012), benefits of anakinra in RA may extend beyond the mere efficacy on articular inflammation and are worth further exploration.

### Gout and Other Forms of Crystal-Induced Arthritis

Monosodium urate crystals activate the NLRP3 inflammasome and induce the release of active IL-1β, with a contribution of free fatty acids, which likely account for the diet-related flares of gout (Joosten et al., 2010). Given the prominent neutrophil infiltration, extracellular processing by neutrophil proteases also likely accounts for activation of IL-1β precursor in gouty joints (Joosten et al., 2009). Traditional options for managing acute flares include colchicine, non-steroidal anti-inflammatory drugs (NSAIDs), and steroids. Treatment with anakinra is dramatically effective at dampening articular inflammation (McGonagle et al., 2007), while also resulting in prolonged periods without flares. Of note, the short half-life and excellent safety profile makes anakinra an ideal therapeutic option for the treatment of acute flares of gouty arthritis, and possibly of patients with underlying chronic kidney disease. Pyrophosphate crystal arthritis, a disease highly reminiscent of gout, is also characteristically responsive to anakinra (McGonagle et al., 2008; Announ et al., 2009), as is another common crystal-induced inflammatory condition, that is, acute calcific periarthritis of the shoulder (Zufferey and So, 2013). In these conditions, short-course anakinra treatment is associated with durable reduction of pain and function impairment, and with normalization of inflammatory indexes.

### Osteoarthritis

There is clinical and experimental evidence that IL-1 is involved in the pathogenesis of osteoarthritis. Thereby, previous studies evaluated the efficacy of direct instillation of anakinra into affected knee joints. Nevertheless, intra-articular injections of anakinra in patients with knee osteoarthritis yielded limited clinical benefit, which did not extend beyond one month from administration (possibly due to short-term persistence of anakinra in the joint space; Chevalier et al., 2005, 2009). Anakinra has demonstrated some efficacy against joint pain and swelling in erosive osteoarthritis of the hand (Bacconnier et al., 2009). IL-1 inhibition with antibodies to the IL-1 receptor has also been evaluated, again with only modest improvement (Cohen et al., 2011). Recent data from the worldwide CANTOS trial supports a role for IL-1β in osteoarthritis. Although this was not the intent of the study, a highly significant reduction in osteoarthritis pain and improved joint function was reported by those treated with canakinumab compared to patients receiving placebo (Ridker et al., 2017). Patients receiving 150 mg of canakinumab fives times each year reported a low incidence of osteoarthritis (1.67 per 100 person-years for placebo versus 1.12 for canakinumab, *p* < 0.001). Supporting evidence to a role for IL-1β in osteoarthritis from the CANTOS database is derived from (i) the large number of patients enrolled world-wide, (ii) the randomized, placebo controlled nature of the trial, and (iii) the specificity of IL-1β neutralization. The demographics of the CANTOS population include age, high BMI, and type 2 diabetes, each of which is characteristic of the osteoarthritis population. Not unexpectedly, there was also a significant reduction in gouty arthritis (Ridker et al., 2017). Although canakinumab treatment was effective in reducing osteoarthritis, systemic treatment with anakinra or canakinumab is an unlikely therapy for the disease.

## Multifactorial Inflammatory Conditions

### Macrophage Activation Syndrome

#### Role of IL-1 and IL-18 in Macrophage Activation Syndrome

Macrophage activation syndrome (MAS), also known as hemophagocytic lympho-histiocytosis (HLH), is a rare, life-threatening condition characterized by a severe hyper-inflammatory state. It is clinically manifested with fever, elevated ferritin, liver enzymes, triglycerides, and pancytopenia due to phagocytosis of bone marrow hematopoietic precursors. Both genetic (familial HLH) and acquired forms of MAS have been described, the latter associated with infection with Epstein–Barr virus, cytomegalovirus, other herpes viruses, and intracellular bacteria, and also of various lymphomas, especially of T-cell lineage. The incidence of MAS is underestimated, as also suggested by new reports of MAS in patients with Ebola virus, parasitic, and influenza infections (Kumar et al., 2014; van der Ven et al., 2015). In addition, patients with rheumatologic conditions, particularly SoJIA and AOSD but also systemic lupus erythematous, Kawasaki disease (KD), or systemic vasculitis can develop MAS (Grom, 2003; Grom et al., 2003; Grom and Mellins, 2011; Janka, 2012).

The pathogenesis of MAS is captivating increasing interest (Schulert and Grom, 2015), and debate is ongoing as to whether MAS is prevalently mediated by IL-1 or IL-18. In the case of familial HLH, gene expression for IL-18 is upregulated in circulating mononuclear cells (Ogilvie et al., 2007; Sumegi et al., 2011), and serum levels of IL-18 are unusually elevated (Honda et al., 2000; Emmenegger et al., 2002; Maeno et al., 2004; Mazodier et al., 2005; Nold et al., 2010). For comparison, levels of circulating IL-18 are below 1 ng/mL in inflammatory diseases such as severe sepsis, but can reach a 20–30 nm/mm range in MAS complicating systemic SoJIA (Larroche and Mouthon, 2004; Fitzgerald et al., 2005; Mazodier et al., 2005; Wada et al., 2013). However, since an IL-18 neutralizing protein [IL-18 binding protein (IL-18BP)] is present in the circulation in health and promptly increases during inflammation, it is critical to determine the levels of free, biologically active IL-18 (Novick et al., 2001). In patients with MAS, free IL-18 significantly correlated with clinical status and biologic markers of MAS, such as anemia, hypertriglyceridemia, and hyperferritinemia, but also with markers of Th1 lymphocyte or macrophage activation, such as IFNγ and soluble receptors for IL-2 and TNFα (Mazodier et al., 2005). A case of MAS due to a mutation in the NLRC4 inflammasome was successfully treated with IL-18BP, whereas anakinra therapy did not effectively reduce the severity of the disease (Canna et al., 2017).

However, IL-1 is responsible for several signs and symptoms of MAS. For example, fever and the increase in ferritin levels are IL-1-mediated, since IL-18 does not cause fever (Gatti et al., 2002; Robertson et al., 2006), does not induce prostaglandins (Lee et al., 2004), and does not induce hepatic acute phase proteins (Stuyt et al., 2005). It should also be noted that IL-1 induces IL-18, and that this mechanism likely plays a critical role in MAS. For example, IL-1 induces the release of constitutively preformed IL-18 precursor from the endothelium (Pomerantz et al., 2001). Elevated levels of IL-18 in MAS likely reflect release from IL-1-activated endothelium, rather than myeloid origin. IL-1-induced myocardial suppression is also mediated by IL-18 (Pomerantz et al., 2001; Toldo et al., 2014). IL-1 mediates fever, hyperferritimia, coagulopathy, and production of IL-18; IL-18 likely mediates hypersplenism, hypertriglyceridemia, hypotension, and elevated IFNγ. With high levels of IL-18-dependent IFNγ, there is macrophage activation in the bone marrow and hemophagocytosis, which characterizes MAS.

#### Anakinra for MAS

Anakinra has primarily been used in MAS due to SoIJA and occasionally to AOSD (Kelly and Ramanan, 2008; Miettunen et al., 2012; Rajasekaran et al., 2014). Although most studies describe favorable results, reports of efficacy are mixed, and include some cases of SoJIA or AOSD who developed MAS while receiving therapy with IL-1 blockade (Colafrancesco et al., 2017). It is possible that development of MAS in these patients simply reflects extreme severity or inadequate control of underlying diseases. Indeed, increasing the dose of anakinra can result in clinical improvement (Sonmez et al., 2018).

Hints of the efficacy of IL-1 blockade in MAS also come from clinical experience with septic shock. Many years ago, anakinra treatment was evaluated in three randomized, placebo-controlled trials of patients with sepsis or septic shock. In nearly 2000 patients enrolled in these trials, anakinra did not reduce overall all-cause mortality. However, recent re-analysis of data from these original studies revealed that a significant benefit of anakinra treatment could be identified in a subset of patients exhibiting a strikingly inflammatory phenotype, which was highly reminiscent of MAS and clinically characterized by cytopenia and elevated ferritin and liver enzymes levels (Shakoory et al., 2016).

### Behcet’s Disease

Behçet’s disease is a rare vasculitis of small- and medium-sized vessels characterized by ocular and cutaneous inflammation, oral and genital ulcers, gastrointestinal or brain vasculitis, and hypercoagulable state. Ocular involvement may cause organ-threatening uveitis and retinal vasculitis. Severe, steroid-resistant disease responds to IL-1 blockers, which can afford dramatic and sustained reversal of intraocular inflammation (Cantarini et al., 2015a,b).

In an open-label pilot study of anti-IL-1β antibodies (gevokizumab) in the treatment of acute eye inflammation, a single dose prompted complete resolution of pan-uveitis and restored normal vision within 4–21 days (Gul et al., 2012).

### Systemic Vasculitides: Kawasaki Disease, Takayasu Arteritis, Giant Cell Arteritis

Kawasaki disease is one of the most common systemic vasculitides and a leading cause of acquired heart disease in children. It typically affects coronary arteries, and residual vascular damage can cause complications later in life, including myocardial infarctions. Classic treatment options include intravenous immunoglobulin (IVIG) and aspirin. Of note, the beneficial anti-inflammatory and therapeutic effects of IVIG in several immune-mediated disorders include a reduction in IL-1 production with an increase in IL-1Ra (Aukrust et al., 1994). Consistently, reports on the efficacy of anakinra treatment indicate that IL-1 plays a pivotal role in the development of vascular damage in KD (Cohen et al., 2012; Dusser and Kone-Paut, 2017; Blonz et al., 2018; Gamez-Gonzalez et al., 2018; Kone-Paut et al., 2018). A prospective trial on anakinra in KD is underway (NCT02179853).

Coronary and peripheral artery inflammation also characterizes large vessel vasculitides Takayasu and giant cell arteritis (Berti et al., 2015; De Luca et al., 2017; Cavalli et al., 2018). Therapies for steroid- and DMARD-refractory large vessel vasculitides are limited. Analysis of temporal artery specimens from GCA patients revealed that IL-1 is highly expressed in inflamed vessels (Hernandez-Rodriguez et al., 2004): accordingly, anakinra treatment dampened systemic and arterial inflammation in two cases refractory to conventional treatment (Ly et al., 2013).

### Histiocytic Disorders

Erdheim–Chester disease (ECD) is a rare form of non-Langerhans histiocytosis, characterized by infiltration of foamy macrophages into multiple tissues (Campochiaro et al., 2015). The disease is typically sustained by activating mutations along the mitogen-activated protein kinase (MAPK) or related pathways in macrophages, which lead to cell activation and consequent production of high levels of pro-inflammatory cytokines and fibrosis of affected tissues (Cavalli et al., 2014; Cangi et al., 2015; Pacini et al., 2018). Clinical manifestations include bone pain, neurological symptoms, retroperitoneal fibrosis, and congestive heart failure (Cavalli et al., 2013a,b; Ferrero et al., 2016; Iurlo et al., 2016; Chiapparini et al., 2018). Anakinra is effective in ameliorating skeletal, cardiac, retroperitoneal, and systemic manifestations (Aouba et al., 2010; Killu et al., 2013; Franconieri et al., 2016; Tomelleri et al., 2018), thus substantiating the central role of macrophages in diseases responsive to IL-1 blockade (Diamond et al., 2014; Berti et al., 2017; Cavalli et al., 2017a). Interestingly, a traditional treatment option for ECD, that is, alpha interferon (IFNα), may exert beneficial effects via induction of IL-1Ra (Tilg et al., 1993) and inhibition of inflammasome activation (Guarda et al., 2011). Modulation of the IL-1 pathway may indeed explain the efficacy of IFNα in the management of ECD, as well as in a spectrum of clinical conditions similarly characterized by the BRAFV600E mutation and activation of the IL-1 pathway, including hairy cell leukemia and melanoma.

### Hearing Loss

#### Hearing Loss in Autoinflammatory Syndromes

Sensorineural deafness is a prominent characteristic of CAPS, a spectrum of conditions caused by activating mutations in NLRP3 leading to deregulated release of active IL-1β (Aganna et al., 2002), which are effectively treated with IL-1 blockade. The first reports of reversal in sensorineural deafness with anakinra came from patients with MWS, a CAPS subtype (Rynne et al., 2006); several other reports followed (Gerard et al., 2007; Kitley et al., 2010; Ahmadi et al., 2011; Klein and Horneff, 2011; Kuemmerle-Deschner et al., 2011b,c, 2013; Eungdamrong et al., 2013; Stew et al., 2013). This reversal in sensorineural deafness with anakinra treatment delineated the unexpected concept that hearing loss in autoinflammatory diseases is due to a reversible chronic inflammatory response, rather than permanent loss of neuronal function. Nevertheless, early treatment with anakinra is more likely to be beneficial (Sibley et al., 2012). Patients with NOMID, a more severe disease also part of the CAPS spectrum, also benefit from anakinra treatment for hearing loss. In pediatric patients with CAPS, early identification in childhood and early anakinra treatment prevents or rescues sensorineural deafness and hearing loss, and results in normal intellectual development into adulthood (Rigante et al., 2006; Hedrich et al., 2008 #7023; Sibley et al., 2012).

#### Anakinra in Autoimmune Hearing Loss

Sensorineural deafness also occurs in vasculitis and autoimmune inner ear disease, and is clinically manifested as rapidly progressive, often irreversible hearing loss. Treatment relies on high-dose glucocorticoids, but many patients are refractory or become unresponsive over time. In these refractory cases, elevated IL-1β was demonstrated in the circulation and in monocyte cultures (Pathak et al., 2011). In an open-label, single-arm, phase I/II clinical trial of anakinra in corticosteroid-resistant autoimmune inner ear disease, 10 patients received treatment for 12 weeks. Of these, seven obtained audiometric improvement, paralleled by reduced IL-1β plasma levels (Vambutas et al., 2014).

### Dry Eye Disease

Dry eye syndrome, or keratoconjunctivitis sicca, is a common, multifactorial disorder of the eye characterized by deficient tear production, excessive tear evaporation, or both. Meibomian gland dysfunction is thought to be the leading cause of this condition, which results in discomfort, visual disturbance, and ocular surface damage. Topical administration of low-dose (2.5%) anakinra proved effective in a randomized clinical trial of 75 dry eye disease patients, which achieved a significant reduction in mean severity score and symptoms (Amparo et al., 2013). It is tempting to envisage similarly favorable results in patients with Sjögren and sicca syndrome.

### Pulmonary Silicosis

Pulmonary silicosis is an occupational disease caused by inhalation of silica crystals. These are not effectively cleared by alveolar macrophages and induce a chronic inflammatory response eventually leading to pulmonary fibrosis and progressive respiratory insufficiency (Leung et al., 2012). Since silica crystals activate the inflammasome and trigger release of active IL-1β (Hornung et al., 2008), a study evaluated anakinra treatment and documented progressive improvement in respiratory symptoms and pulmonary inflammation in a patient with pulmonary silicosis and severe respiratory failure (Cavalli et al., 2015a).

### Organ Transplant

Previous studies identified associations between levels of IL-1β and IL-1Ra in serum and urine and negative graft outcome; studies in experimental animals and observations in humans also substantiated a possible protective effect of blocking IL-1 after solid organ transplantation (reviewed in Mulders-Manders et al., 2017). New evidence suggests that in patients undergoing solid organ transplantation, IL-1 inhibition in addition to standard immunosuppressive regimens may dampen inflammation and protect against negative graft outcome. Three patients undergoing renal transplantation were receiving treatment with anakinra in the peri-operative and post-operative period for underlying IL-1-driven autoinflammatory diseases (AOSD, CAPS, and FMF, respectively). Kidney function increased rapidly in all patients; anakinra was well tolerated and safe with the exception of minor infections (Mulders-Manders et al., 2017). The beneficial effects of treatment are likely due to dampening of ischemia-reperfusion injury, which accompanies renal transplantation and leads to release of IL-1 and to impaired graft function.

## Anakinra for Central Nervous System Diseases

Neurologic complications observed in CAPS patients reveal the effects of IL-1-mediated inflammation in the brain. Common clinical manifestations include headache or migraine, sensory-neural hearing loss, papilledema due to elevated intracranial pressure, and mental impairment (Kitley et al., 2010). IL-1 blockade with anakinra or canakinumab reverses neurologic inflammation and related symptoms, including mental and hearing impairment (Lachmann et al., 2009; Goldbach-Mansky, 2011; Kuemmerle-Deschner et al., 2011a; Lepore et al., 2011; Neven et al., 2011).

### Anakinra Enters the Brain

The first evidence that anakinra administered peripherally crossed the blood–brain barrier and reduced severity of a disease primarily localized to the central nervous system came from NOMID (Goldbach-Mansky et al., 2006). Specifically, 12 children with NOMID were treated with 1–2 mg/kg of subcutaneous anakinra daily. The median cerebrospinal fluid (CSF) level of IL-1Ra was 211 pg/mL before treatment, but rose to 1136 pg/mL after 3 months of treatment (Goldbach-Mansky et al., 2006). These effects were associated with a remarkable decrease in the severity of various NOMID manifestations, including elevated intracranial pressure, leptomeningitis, and neurosensorial hearing loss, as well as reduced CSF levels of IL-6.

Intravenous anakinra was also administered to patients with subarachnoid hemorrhage due to aneurysmal rupture (Singh et al., 2014), again in a placebo-controlled setting. Within 72 h of the acute event, patients received a bolus infusion of 500 mg of anakinra followed by a steady infusion of 10 mg/kg/h for 24 h. At 24 h, CSF levels of IL-6 were reduced in the anakinra compared to the placebo group (Singh et al., 2014).

A related study investigated the dose regimen necessary to obtain a CSF concentration of anakinra 100 ng/mL. This concentration (100 ng/mL) was deemed neuro-protective based on studies of rats subjected to brain ischemia (Clark et al., 2008); as for human reference, this target concentration of 100 ng/mL is 100-fold greater than that in the CSF of children receiving subcutaneous anakinra 100 mg daily for 3 months (Goldbach-Mansky et al., 2006). In this study, patients with subarachnoid bleed received incremental doses of intravenous anakinra (Galea et al., 2011): specifically, the patients received a bolus dose of anakinra (100–500 mg) followed by a 4-h infusion of anakinra from 1 to 10 mg/kg/h. Levels of anakinra were monitored in plasma and CSF (collected through a cerebral ventricular drain). A target CSF level of 100 ng/mL was achieved with the highest regimen (a bolus of 500 mg followed by 4 h of anakinra at 10 mg/kg/h; Galea et al., 2011; Ogungbenro et al., 2016). The authors concluded that anakinra passively enters the brain in patients with a subarachnoid hemorrhage: therefore, a high-dose regimen of anakinra may reduce inflammation, infiltration of neutrophils, and edema at the site of the lesion. In a subsequent randomized study, patients with subarachnoid hemorrhage received anakinra 100 mg twice daily subcutaneously within 3 days of stroke and for the following 21 days. Again, anakinra treatment significantly reduced levels of inflammatory markers IL-6, CRP, and fibrinogen. Although these studies were not powered to determine clinical effects, scores of the Glasgow Outcome Scale at 6 months were better, albeit not significantly, among patients receiving anakinra. Whether dampening of IL-1-mediated inflammation will result in improved neurological outcomes remains to be determined in adequately powered, randomized, placebo-controlled studies.

In a different study, intravenous anakinra was administered to patients admitted to the hospital within 6 h of an acute thrombotic stroke (Emsley et al., 2005). This trial included 34 patients and was randomized and placebo controlled; anakinra was administered at a high dose of 2 mg/kg/h for 72 h, analogous to inception trials of anakinra in septic shock. Compared to placebo-treated controls, patients treated with anakinra had lower IL-6, CRP, and neutrophil levels (Emsley et al., 2005). Although the study was not powered for detecting significant improvements in neurological outcomes, the subgroup of patients with cortical infarcts receiving anakinra performed better compared to the placebo group.

Additional evidence that anakinra crosses the blood brain barrier and exerts anti-inflammatory effects in the brain comes from studies of traumatic brain injury, a major cause of death and disability worldwide, particularly in young persons. In a randomized, open-label trial, 20 patients who had suffered diffuse traumatic brain injury within the previous 24 h received either anakinra 100 mg daily for 5 days or placebo. A central microdialysis catheter was placed in each patient as part of standard of care. Prior to administration of anakinra, the mean level of IL-1Ra in the CSF was 78 pg/mL but rose to 138 pg/mL 12 h after the first dose (Helmy et al., 2014). In general, inflammatory cytokines in the CSF were lower in patients treated with anakinra; of these, macrophage-derived chemoattractant-1 (MDC-1) was remarkably lower compared to patients treated with the placebo (1.04 pg/mL in the anakinra group compared to 45.4 pg/mL in the placebo group; Helmy et al., 2014). The study was not powered to evaluate clinical improvement, although the marked decrease in CSF levels of cytokines and MDC-1 argue in favor of beneficial anti-inflammatory effects.

### Epilepsy

Although IL-1α is found in brain astrocytes and microglia, available data point at IL-1β as the main contributor to epileptic seizures (Vezzani et al., 2011). Several studies have focused on febrile seizures since these are among the most common type of seizure activity. Using an animal model for febrile seizures, an agonist role for IL-1β and an antagonist role for endogenous IL-1Ra in the hippocampus have been reported (Heida et al., 2009). Other studies examined circulating cytokines in patients with recurrent seizures, and revealed elevated levels of IL-6 and IL-1Ra in the post-acute period (Uludag et al., 2013). In one study, high levels of IL-1β were also observed during acute episodes of recurrent temporal lobe epilepsy (Uludag et al., 2015). Some studies have reported polymorphisms in IL-1α, IL-1β, and IL-1Ra in subjects who develop epilepsy as adults (Kanemoto et al., 2000; Haspolat et al., 2005; Nakayama and Arinami, 2006; Serdaroglu et al., 2009; Chou et al., 2010). In experimental animals, suppression of peripheral IL-1-mediated inflammation reduces the severity of status epilepticus (Marchi et al., 2009).

Anakinra has been administered to a young patient with a severe seizure disorder termed febrile infection-related epilepsy syndrome (FIRES; Hirsch et al., 2018). This syndrome, which often follows an infectious encephalopathy, has a high mortality rate and few treatment options. This patient had recurrent seizures each day, which progressively decreased in frequency and eventually ceased while being treated with daily subcutaneous anakinra (Kenney-Jung et al., 2016). This was mirrored by a decrease in CSF pro-inflammatory cytokines. When anakinra was stopped, seizures resumed only to decrease again upon restarting. A subsequent study confirmed the favorable outcome with anakinra described in this report. Anakinra was administered to five children with FIRES and refractory status epilepticus beginning shortly after a febrile illness. All had received anti-epileptic drugs (AEDs, ranging from two to six different medications), had required anesthetics for seizure control, and had received treatment with corticosteroids and IVIG; three underwent plasmapheresis. Anakinra was initiated on day 12–32 of illness at a dose ranging from 3 to 7 mg/kg/day, and led to rapid and remarkable clinical improvement in all but one patient. Specifically, seizure count in the week prior to anakinra initiation ranged from 8 to 170, but dropped to 0–12 after 1 week of anakinra treatment and to 0–7 after 4 weeks of treatment. Two patients had increased seizure burden upon anakinra weaning or discontinuation, again substantiating the role of IL-1 in seizure disorder (Shukla et al., 2018). In another study, an adolescent female with signs of persistent systemic inflammation and epilepsy unresponsive to multiple AEDs also promptly responded to anakinra (DeSena et al., 2018).

## Safety of Il-1 Blockade With Anakinra

Impaired host defense against pathogens is a concern for cytokine-blocking agents. In patients treated with biologics, particularly TNFα blocking therapies, there is an increased risk of several opportunistic infections, similar to those observed in immunosuppressed persons. Host defense against opportunistic organisms as well as common bacterial infections have since become a major concern for all anti-cytokine agents because of the indolent and dangerous nature of these infections. For example, reactivation of latent *M. tuberculosis* in patients receiving anti-TNFα therapies can be 25 times higher than in untreated persons (Solovic et al., 2011) and is often in the disseminated form, similar to that observed in HIV-1 infected patients. *M. tuberculosis* also occurs in patients treated with TNFα blockers without evidence of prior exposure to the organism. Despite screening for previous exposure to *M. tuberculosis* before beginning any anti-cytokine treatment, reactivation continues to occur and can be as high as 9.3% (Chiu et al., 2011).

As with all biologic agents, an increase in infection frequency has been reported for anakinra. Nevertheless, in comparison to other biologic agents, anakinra has an unparalleled safety benefit deriving from short half-life and effect duration, and has demonstrated a remarkable record of safety (Fleischmann et al., 2006; Mertens and Singh, 2009b). Since introduction in 2002, it is estimated that over 150,000 patients have received anakinra, some treated daily for over 10 years. Opportunistic infections in patients treated with anakinra are rare (Fleischmann et al., 2003), including in populations at high risk for reactivation of *M. tuberculosis* infections (Bresnihan et al., 1998; Lopalco et al., 2016a,b). There is a single case report of a 77-year-old man with severe RA and a history of pulmonary tuberculosis who developed a reactivation 23 months after starting anakinra (Settas et al., 2007).

In addition, a large number of animal studies including primates subjected to live bacteria inoculum demonstrated greater survival in infected animals treated with anakinra compared to vehicle. In humans, anakinra has been administered to patients with active infections (Hennig et al., 2010; van de Veerdonk et al., 2011), and in over 2000 patients in trials of sepsis and septic shock without any increase in mortality despite exceedingly high dosing (30-fold higher than the current approved dose of 100 mg/day; Dinarello et al., 2012). Other safety examples include hidradenitis suppurativa, in which anakinra treatment resolves inflammation of *Staphylococcus aureus*-infected apocrine glands (Braun-Falco et al., 2011; Hsiao et al., 2011; Zarchi et al., 2013; Tzanetakou et al., 2016), and chronic granulomatous disease, an inherited condition with recurrent bouts of infections with Gram-positive and Gram-negative bacteria as well as fungi (van de Veerdonk et al., 2011): in both conditions, treatment with anakinra reduces the severity of inflammation without increasing the infection burden.

During controlled trials of anakinra, there were more viral-type upper airway infections compared to placebo-treated patients, as for most other biologics. There are two spurious reports of anakinra-related hepatotoxicity in patients with AOSD; however, withdrawal of anakinra restored normal liver function. Of note, there are several reports of the safety of increasing the dose of anakinra to 200 mg/day or above (Vitale et al., 2016; Colafrancesco et al., 2017; Grayson et al., 2017).

Subcutaneous administrations of anakinra often cause injection site reactions. Albeit uncomfortable due to the need for daily injections, these usually resolve within 2–3 weeks of treatment initiation. A fraction of patients receiving treatment with anakinra can develop antibodies against the drug (Cohen et al., 2002; Ilowite et al., 2009). As with other biologic agents, the potential for adverse effects including hypersensitivity reactions or aplasia should be carefully monitored. Conversely, these antibodies are usually non-neutralizing and do not decrease the biologic effectiveness of anakinra, nor they appear to be linked to the development of injection site reaction.

Interleukin-1 injected into humans at doses as low as 3 ng/kg induce neutrophil mobilization from the bone marrow and neutrophilia (Dinarello, 1996; Ogilvie et al., 1996). Hence, most patients with IL-1-mediated conditions exhibit neutrophilia as a hematological manifestation of their disease. A reduction in circulating neutrophils upon anakinra administration can be observed and often heralds a clinical response; sustained neutropenia is not typically observed, but neutrophil levels occasionally fall below the normal range, only to rise rapidly upon cessation of treatment (Cavalli and Dinarello, 2015).

## Author Contributions

GC and CD wrote the manuscript.

## Conflict of Interest Statement

The authors declare that the research was conducted in the absence of any commercial or financial relationships that could be construed as a potential conflict of interest.

## References

[B1] Abbate A. Kontos M. C. Grizzard J. D. Biondi-Zoccai G. G. Van Tassell B. W. Robati R. (2010). Interleukin-1 blockade with anakinra to prevent adverse cardiac remodeling after acute myocardial infarction (Virginia Commonwealth University Anakinra Remodeling Trial [VCU-ART] Pilot study). *Am. J. Cardiol.* 105 1371.e1–1377.e1. 10.1016/j.amjcard.2009.12.059 20451681

[B2] Abbate A. Van Tassell B. W. Biondi-Zoccai G. Kontos M. C. Grizzard J. D. Spillman D. W. (2013). Effects of interleukin-1 blockade with anakinra on adverse cardiac remodeling and heart failure after acute myocardial infarction [from the Virginia Commonwealth University-Anakinra Remodeling Trial (2) (VCU-ART2) pilot study]. *Am. J. Cardiol.* 111 1394–1400. 10.1016/j.amjcard.2013.01.287 23453459 PMC3644511

[B3] Aganna E. Martinon F. Hawkins P. N. Ross J. B. Swan D. C. Booth D. R. (2002). Association of mutations in the NALP3/CIAS1/PYPAF1 gene with a broad phenotype including recurrent fever, cold sensitivity, sensorineural deafness, and AA amyloidosis. *Arthritis Rheum.* 46 2445–2452. 12355493 10.1002/art.10509

[B4] Agostini L. Martinon F. Burns K. McDermott M. F. Hawkins P. N. Tschopp J. (2004). NALP3 forms an IL-1beta processing inflammasome with increased activity in muckle-wells auto-inflammatory disorder. *Immunity* 20 319–325. 15030775 10.1016/s1074-7613(04)00046-9

[B5] Ahmadi N. Brewer C. C. Zalewski C. King K. A. Butman J. A. Plass N. (2011). Cryopyrin-associated periodic syndromes: otolaryngologic and audiologic manifestations. *Otolaryngol. Head Neck Surg.* 145 295–302. 10.1177/0194599811402296 21493283 PMC3407887

[B6] Ait-Abdesselam T. Lequerre T. Legallicier B. Francois A. Le Loet X. Vittecoq O. (2011). Anakinra efficacy in a Caucasian patient with renal AA amyloidosis secondary to cryopyrin-associated periodic syndrome. *Joint Bone Spine* 77 616–617. 20609610 10.1016/j.jbspin.2010.04.018

[B7] Aksentijevich I. Masters S. L. Ferguson P. J. Dancey P. Frenkel J. van Royen-Kerkhoff A. (2009). An autoinflammatory disease with deficiency of the interleukin-1-receptor antagonist. *N. Engl. J. Med.* 360 2426–2437. 10.1056/NEJMoa0807865 19494218 PMC2876877

[B8] Alten R. Gomez-Reino J. Durez P. Beaulieu A. Sebba A. Krammer G. (2011). Efficacy and safety of the human anti-IL-1beta monoclonal antibody canakinumab in rheumatoid arthritis: results of a 12-week, phase II, dose-finding study. *BMC Musculoskelet. Disord.* 12:153. 10.1186/1471-2474-12-153 21736751 PMC3152943

[B9] Ambrose N. L. O’Connell P. G. (2007). Anti-TNF alpha therapy does not always protect rheumatoid arthritis patients against developing pericarditis. *Clin. Exp. Rheumatol.* 25:660. 17888231

[B10] Amparo F. Dastjerdi M. H. Okanobo A. Ferrari G. Smaga L. Hamrah P. (2013). Topical interleukin 1 receptor antagonist for treatment of dry eye disease: a randomized clinical trial. *JAMA Ophthalmol.* 131 715–723. 10.1001/jamaophthalmol.2013.195 23599118 PMC4167802

[B11] Announ N. Palmer G. Guerne P. A. Gabay C. (2009). Anakinra is a possible alternative in the treatment and prevention of acute attacks of pseudogout in end-stage renal failure. *Joint Bone Spine* 76 424–426. 10.1016/j.jbspin.2009.01.001 19289295

[B12] Aouba A. Georgin-Lavialle S. Pagnoux C. Martin Silva N. Renand A. Galateau-Salle F. (2010). Rationale and efficacy of interleukin-1 targeting in Erdheim-Chester disease. *Blood* 116 4070–4076. 10.1182/blood-2010-04-279240 20724540

[B13] Arend W. P. Joslin F. G. Massoni R. J. (1985). Effects of immune complexes on production by human monocytes of interleukin 1 or an interleukin 1 inhibitor. *J. Immunol.* 134 3868–3875. 2985700

[B14] Aronson I. K. Worobec S. M. (2010). Cytophagic histiocytic panniculitis and hemophagocytic lymphohistiocytosis: an overview. *Dermatol. Ther.* 23 389–402. 10.1111/j.1529-8019.2010.01339.x 20666826

[B15] Arostegui J. I. Arnal C. Merino R. Modesto C. Antonia Carballo M. Moreno P. (2007). NOD2 gene-associated pediatric granulomatous arthritis: clinical diversity, novel and recurrent mutations, and evidence of clinical improvement with interleukin-1 blockade in a Spanish cohort. *Arthritis Rheum.* 56 3805–3813. 17968944 10.1002/art.22966

[B16] Aukrust P. Froland S. S. Liabakk N. B. Muller F. Nordoy I. Haug C. (1994). Release of cytokines, soluble cytokine receptors, and interleukin-1 receptor antagonist after intravenous immunoglobulin administration in vivo. *Blood* 84 2136–2143. 7919327

[B17] Auron P. E. Webb A. C. Rosenwasser L. J. Mucci S. F. Rich A. Wolff S. M. (1984). Nucleotide sequence of human monocyte interleukin 1 precursor cDNA. *Proc. Natl. Acad. Sci. U.S.A.* 81 7907–7911. 6083565 10.1073/pnas.81.24.7907 PMC392262

[B18] Bacconnier L. Jorgensen C. Fabre S. (2009). Erosive osteoarthritis of the hand: clinical experience with anakinra. *Ann. Rheum. Dis.* 68 1078–1079. 19435727 10.1136/ard.2008.094284

[B500] Balavoine J. F. de Rochemonteix B. Williamson K. Seckinger P. Cruchaud A. Dayer J.-M. (1984). Identification of interleukin 1-like activity and inhibitor(s) in urine from a patient with acute monoblastic leukemia. *Lymphokine Res*. 3:23.

[B501] Balavoine J. F. de Rochemonteix B. Williamson K. Seckinger P. Cruchaud A. Dayer J. M. (1986). Prostaglandin E2 and collagenase production by fibroblasts and synovial cells is regulated by urine-derived human interleukin 1 and inhibitor(s). *J. Clin. Invest.* 78 1120–1124. 10.1172/JCI112669 3020090 PMC423775

[B19] Ballak D. B. Li S. Cavalli G. Stahl J. L. Tengesdal I. W. van Diepen J. A. (2018). Interleukin-37 treatment of mice with metabolic syndrome improves insulin sensitivity and reduces pro-inflammatory cytokine production in adipose tissue. *J. Biol. Chem.* 293 14224–14236. 10.1074/jbc.RA118.003698 30006351 PMC6139546

[B20] Behrens E. M. Kreiger P. A. Cherian S. Cron R. Q. (2006). Interleukin 1 receptor antagonist to treat cytophagic histiocytic panniculitis with secondary hemophagocytic lymphohistiocytosis. *J. Rheumatol.* 33 2081–2084. 17014024

[B21] Belani H. Gensler L. Bajpai U. Meinhardt E. Graf J. Pincus L. (2013). Neutrophilic urticaria with systemic inflammation: a case series. *JAMA Dermatol.* 149 453–458. 10.1001/jamadermatol.2013.2705 23715523

[B22] Berti A. Campochiaro C. Cavalli G. Pepe G. Praderio L. Sabbadini M. G. (2015). Giant cell arteritis restricted to the limb arteries: an overlooked clinical entity. *Autoimmun. Rev.* 14 352–357. 10.1016/j.autrev.2014.12.005 25528219

[B23] Berti A. Cavalli G. Guglielmi B. Biavasco R. Campochiaro C. Tomelleri A. (2017). Tocilizumab in patients with multisystem erdheim-chester disease. *Oncoimmunology* 6:e1318237. 10.1080/2162402X.2017.1318237 28680751 PMC5486186

[B24] Blonz G. Lacroix S. Benbrik N. Warin-Fresse K. Masseau A. Trewick D. (2018). Severe late-onset kawasaki disease successfully treated with anakinra. *J. Clin. Rheumatol.* 10.1097/RHU.0000000000000814 [Epub ahead of print]. 32073531

[B25] Bodar E. J. Kuijk L. M. Drenth J. P. van der Meer J. W. Simon A. Frenkel J. (2011). On-demand anakinra treatment is effective in mevalonate kinase deficiency. *Ann. Rheum. Dis.* 70 2155–2158. 10.1136/ard.2011.149922 21859689

[B26] Boni-Schnetzler M. Hauselmann S. P. Dalmas E. Meier D. T. Thienel C. Traub S. (2018). beta cell-specific deletion of the IL-1 receptor antagonist impairs beta cell proliferation and insulin secretion. *Cell Rep.* 22 1774–1786. 10.1016/j.celrep.2018.01.063 29444430

[B27] Botsios C. Sfriso P. Furlan A. Ostuni P. Biscaro M. Fiocco U. (2007). Anakinra, a recombinant human IL-1 receptor antagonist, in clinical practice. Outcome in 60 patients with severe rheumatoid arthritis. *Reumatismo* 59 32–37. 17435840 10.4081/reumatismo.2007.32

[B28] Boyer E. M. Turman M. O’Neil K. M. (2011). Partial response to anakinra in life-threatening Henoch-Schonlein purpura: case report. *Pediatr. Rheumatol. J.* 9:21. 10.1186/1546-0096-9-21 21834965 PMC3169465

[B29] Braun-Falco M. Kovnerystyy O. Lohse P. Ruzicka T. (2011). Pyoderma gangrenosum, acne, and suppurative hidradenitis (PASH)-a new autoinflammatory syndrome distinct from PAPA syndrome. *J. Am. Acad. Dermatol.* 66 409–415. 10.1016/j.jaad.2010.12.025 21745697

[B30] Brenner M. Ruzicka T. Plewig G. Thomas P. Herzer P. (2009). Targeted treatment of pyoderma gangrenosum in PAPA (pyogenic arthritis, pyoderma gangrenosum and acne) syndrome with the recombinant human interleukin-1 receptor antagonist anakinra. *Br. J. Dermatol.* 161 1199–1201. 10.1111/j.1365-2133.2009.09404.x 19673875

[B31] Bresnihan B. Alvaro-Gracia J. M. Cobby M. Doherty M. Domljan Z. Emery P. (1998). Treatment of rheumatoid arthritis with recombinant human interleukin-1 receptor antagonist. *Arthritis Rheum.* 41 2196–2204. 9870876 10.1002/1529-0131(199812)41:12<2196::AID-ART15>3.0.CO;2-2

[B32] Bresnihan B. Newmark R. Robbins S. Genant H. K. (2004). Effects of anakinra monotherapy on joint damage in patients with rheumatoid arthritis. Extension of a 24-week randomized, placebo-controlled trial. *J. Rheumatol.* 31 1103–1111. 15170922

[B33] Brucato A. Imazio M. Gattorno M. Lazaros G. Maestroni S. Carraro M. (2016). Effect of anakinra on recurrent pericarditis among patients with colchicine resistance and corticosteroid dependence: the AIRTRIP randomized clinical Trial. *JAMA* 316 1906–1912. 10.1001/jama.2016.15826 27825009

[B34] Buckley L. F. Abbate A. (2018). Interleukin-1 blockade in cardiovascular diseases: a clinical update. *Eur. Heart J.* 39 2063–2069. 10.1093/eurheartj/ehy128 29584915

[B35] Cain B. S. Meldrum D. R. Dinarello C. A. Meng X. Joo K. S. Banerjee A. (1999). Tumor necrosis factor-a and interleukin-1b synergistically depress human myocardial function. *Crit. Care Med.* 27 1309–1318. 10446825 10.1097/00003246-199907000-00018

[B36] Campochiaro C. Tomelleri A. Cavalli G. Berti A. Dagna L. (2015). Erdheim-chester disease. *Eur. J. Intern. Med.* 26 223–229. 10.1016/j.ejim.2015.03.004 25865950

[B37] Cangi M. G. Biavasco R. Cavalli G. Grassini G. Dal-Cin E. Campochiaro C. (2015). BRAFV600E-mutation is invariably present and associated to oncogene-induced senescence in Erdheim-Chester disease. *Ann. Rheum. Dis.* 74 1596–1602. 10.1136/annrheumdis-2013-204924 24671772

[B38] Canna S. W. Girard C. Malle L. de Jesus A. Romberg N. Kelsen J. (2017). Life-threatening NLRC4-associated hyperinflammation successfully treated with IL-18 inhibition. *J. Allergy Clin. Immunol.* 139 1698–1701. 10.1016/j.jaci.2016.10.022 27876626 PMC5846100

[B39] Cannon J. G. Dinarello C. A. (1985). Increased plasma interleukin-1 activity in women after ovulation. *Science* 227 1247–1249. 3871966 10.1126/science.3871966

[B40] Cantarini L. Lopalco G. Caso F. Costa L. Iannone F. Lapadula G. (2015a). Effectiveness and tuberculosis-related safety profile of interleukin-1 blocking agents in the management of Behcet’s disease. *Autoimmun. Rev.* 14 1–9. 10.1016/j.autrev.2014.08.008 25151975

[B41] Cantarini L. Vitale A. Scalini P. Dinarello C. A. Rigante D. Franceschini R. (2015b). Anakinra treatment in drug-resistant Behcet’s disease: a case series. *Clin. Rheumatol.* 34 1293–1301. 10.1007/s10067-013-2443-8 24305945

[B42] Cantarini L. Lucherini O. M. Cimaz R. Galeazzi M. (2010). Recurrent pericarditis caused by a rare mutation in the TNFRSF1A gene and with excellent response to anakinra treatment. *Clin. Exp. Rheumatol.* 28:802. 21029567

[B43] Cantarini L. Lucherini O. M. Cimaz R. Rigante D. Baldari C. T. Laghi Pasini F. (2012a). Typical and severe tumor necrosis factor receptor-associated periodic syndrome in the absence of mutations in the TNFRSF1A gene: a case series. *Rheumatol. Int.* 32 4015–4018. 10.1007/s00296-010-1512-4 20473499

[B44] Cantarini L. Lucherini O. M. Muscari I. Frediani B. Galeazzi M. Brizi M. G. (2012b). Tumour necrosis factor receptor-associated periodic syndrome (TRAPS): state of the art and future perspectives. *Autoimmun. Rev.* 12 38–43. 10.1016/j.autrev.2012.07.020 22884554

[B45] Cavalli G. Berti A. Campochiaro C. Dagna L. (2013a). Diagnosing erdheim-chester disease. *Ann. Rheum. Dis.* 72:e19. 10.1136/annrheumdis-2013-203685 23592711

[B46] Cavalli G. Guglielmi B. Berti A. Campochiaro C. Sabbadini M. G. Dagna L. (2013b). The multifaceted clinical presentations and manifestations of Erdheim-Chester disease: comprehensive review of the literature and of 10 new cases. *Ann. Rheum. Dis.* 72 1691–1695. 10.1136/annrheumdis-2012-202542 23396641

[B47] Cavalli G. Biavasco R. Borgiani B. Dagna L. (2014). Oncogene-induced senescence as a new mechanism of disease: the paradigm of erdheim-chester disease. *Front. Immunol.* 5:281. 10.3389/fimmu.2014.00281 24982657 PMC4056107

[B48] Cavalli G. De Luca G. Dagna L. (2017a). Advances in potential targeted therapies for erdheim-chester disease. *Expert Opin. Orphan Drugs* 5 253–260. 10.1080/21678707.2017.1285226 28598870

[B49] Cavalli G. Foppoli M. Cabrini L. Dinarello C. A. Tresoldi M. Dagna L. (2017b). Interleukin-1 Receptor blockade rescues myocarditis-associated end-stage heart failure. *Front. Immunol.* 8:131. 10.3389/fimmu.2017.00131 28232838 PMC5298961

[B50] Cavalli G. Justice J. N. Boyle K. E. D’Alessandro A. Eisenmesser E. Z. Herrera J. J. (2017c). Interleukin 37 reverses the metabolic cost of inflammation, increases oxidative respiration, and improves exercise tolerance. *Proc. Natl. Acad. Sci. U.S.A.* 114 2313–2318. 10.1073/pnas.1619011114 28193888 PMC5338542

[B51] Cavalli G. Koenders M. Kalabokis V. Kim J. Choon Tan A. Garlanda C. (2017d). Treating experimental arthritis with the innate immune inhibitor interleukin-37 reduces joint and systemic inflammation. *Rheumatology* 56 2220–2229. 10.1093/rheumatology/kex348 27567100 PMC5144668

[B52] Cavalli G. Dinarello C. A. (2015). Treating rheumatological diseases and co-morbidities with interleukin-1 blocking therapies. *Rheumatology* 54 2134–2144. 10.1093/rheumatology/kev269 26209330 PMC5009422

[B53] Cavalli G. Dinarello C. A. (2018). Suppression of inflammation and acquired immunity by IL-37. *Immunol. Rev.* 281 179–190. 10.1111/imr.12605 29247987

[B54] Cavalli G. Fallanca F. Dinarello C. A. Dagna L. (2015a). Treating pulmonary silicosis by blocking interleukin 1. *Am. J. Respir. Crit. Care Med.* 191 596–598. 10.1164/rccm.201412-2150LE 25723826

[B55] Cavalli G. Franchini S. Aiello P. Guglielmi B. Berti A. Campochiaro C. (2015b). Efficacy and safety of biological agents in adult-onset Still’s disease. *Scand. J. Rheumatol.* 44 309–314. 10.3109/03009742.2014.992949 25656459

[B56] Cavalli G. Hayashi M. Jin Y. Yorgov D. Santorico S. A. Holcomb C. (2016a). MHC class II super-enhancer increases surface expression of HLA-DR and HLA-DQ and affects cytokine production in autoimmune vitiligo. *Proc. Natl. Acad. Sci. U.S.A.* 113 1363–1368. 10.1073/pnas.1523482113 26787888 PMC4747741

[B57] Cavalli G. Koenders M. Kalabokis V. Kim J. Tan A. C. Garlanda C. (2016b). Treating experimental arthritis with the innate immune inhibitor interleukin-37 reduces joint and systemic inflammation. *Rheumatology* 55 2220–2229. 10.1093/rheumatology/kew325 27567100 PMC5144668

[B58] Cavalli G. Pappalardo F. Mangieri A. Dinarello C. A. Dagna L. Tresoldi M. (2016c). Treating life-threatening myocarditis by blocking interleukin-1. *Crit. Care Med.* 44 e751–e754. 10.1097/CCM.0000000000001654 27031379

[B59] Cavalli G. Tomelleri A. Di Napoli D. Baldissera E. Dagna L. (2018). Prevalence of Takayasu arteritis in young women with acute ischemic heart disease. *Int. J. Cardiol.* 252 21–23. 10.1016/j.ijcard.2017.10.067 29249430

[B60] Cavelti-Weder C. Babians-Brunner A. Keller C. Stahel M. A. Kurz-Levin M. Zayed H. (2012). Effects of gevokizumab on glycemia and inflammatory markers in type 2 diabetes. *Diabetes Care* 35 1654–1662. 10.2337/dc11-2219 22699287 PMC3402269

[B61] Chae J. J. Wood G. Masters S. L. Richard K. Park G. Smith B. J. (2006). The B30.2 domain of pyrin, the familial Mediterranean fever protein, interacts directly with caspase-1 to modulate IL-1beta production. *Proc. Natl. Acad. Sci. U.S.A.* 103 9982–9987. 16785446 10.1073/pnas.0602081103 PMC1479864

[B62] Chevalier X. Giraudeau B. Conrozier T. Marliere J. Kiefer P. Goupille P. (2005). Safety study of intraarticular injection of interleukin 1 receptor antagonist in patients with painful knee osteoarthritis: a multicenter study. *J. Rheumatol.* 32 1317–1323. 15996071

[B63] Chevalier X. Goupille P. Beaulieu A. D. Burch F. X. Bensen W. G. Conrozier T. (2009). Intraarticular injection of anakinra in osteoarthritis of the knee: a multicenter, randomized, double-blind, placebo-controlled study. *Arthritis Rheum.* 61 344–352. 10.1002/art.24096 19248129

[B64] Chiapparini L. Cavalli G. Langella T. Venerando A. De Luca G. Raspante S. (2018). Adult leukoencephalopathies with prominent infratentorial involvement can be caused by Erdheim-Chester disease. *J. Neurol.* 265 273–284. 10.1007/s00415-017-8692-8 29204962

[B65] Chiu H. Y. Hsueh P. R. Tsai T. F. (2011). Clinical experience of QuantiFERON((R)) -TB Gold testing in patients with psoriasis treated with tumour necrosis factor blockers in Taiwan. *Br. J. Dermatol.* 164 553–559. 10.1111/j.1365-2133.2010.10137.x 21083541

[B66] Choi A. D. Moles V. Fuisz A. Weissman G. (2014). Cardiac magnetic resonance in myocarditis from adult onset Still’s disease successfully treated with anakinra. *Int. J. Cardiol.* 172 e225–e227. 10.1016/j.ijcard.2013.12.151 24461482

[B67] Chou I. C. Lin W. D. Wang C. H. Tsai C. H. Li T. C. Tsai F. J. (2010). Interleukin (IL)-1beta, IL-1 receptor antagonist, IL-6, IL-8, IL-10, and tumor necrosis factor alpha gene polymorphisms in patients with febrile seizures. *J. Clin. Lab. Anal.* 24 154–159. 10.1002/jcla.20374 20486195 PMC6647684

[B68] Clark S. R. McMahon C. J. Gueorguieva I. Rowland M. Scarth S. Georgiou R. (2008). Interleukin-1 receptor antagonist penetrates human brain at experimentally therapeutic concentrations. *J. Cereb. Blood Flow Metab.* 28 387–394. 10.1038/sj.jcbfm.9600537 17684519

[B69] Cohen S. Hurd E. Cush J. Schiff M. Weinblatt M. E. Moreland L. W. (2002). Treatment of rheumatoid arthritis with anakinra, a recombinant human interleukin-1 receptor antagonist, in combination with methotrexate: results of a twenty-four-week, multicenter, randomized, double-blind, placebo-controlled trial. *Arthritis Rheum.* 46 614–624. 10.1002/art.10141 11920396

[B70] Cohen S. Tacke C. E. Straver B. Meijer N. Kuipers I. M. Kuijpers T. W. (2012). A child with severe relapsing Kawasaki disease rescued by IL-1 receptor blockade and extracorporeal membrane oxygenation. *Ann. Rheum. Dis.* 71 2059–2061. 10.1136/annrheumdis-2012-201658 22689319

[B71] Cohen S. B. Proudman S. Kivitz A. J. Burch F. X. Donohue J. P. Burstein D. (2011). A randomized, double-blind study of AMG 108 (a fully human monoclonal antibody to IL 1R1) in patients with osteoarthritis of the knee. *Arthritis Res. Ther.* 13:R125. 10.1186/ar3430 21801403 PMC3239365

[B72] Colafrancesco S. Priori R. Valesini G. Argolini L. Baldissera E. Bartoloni E. (2017). Response to interleukin-1 inhibitors in 140 Italian patients with adult-onset Still’s disease: a multicentre retrospective observational study. *Front. Pharmacol.* 8:369 10.3389/fphar.2017.00369 PMC5469286 28659802

[B73] Colina M. Pizzirani C. Khodeir M. Falzoni S. Bruschi M. Trotta F. (2010). Dysregulation of P2X7 receptor-inflammasome axis in SAPHO syndrome: successful treatment with anakinra. *Rheumatology* 49 1416–1418. 20299381 10.1093/rheumatology/keq074

[B74] de Koning H. D. Schalkwijk J. van der Ven-Jongekrijg J. Stoffels M. van der Meer J. W. Simon A. (2013). Sustained efficacy of the monoclonal anti-interleukin-1 beta antibody canakinumab in a 9-month trial in Schnitzler’s syndrome. *Ann. Rheum. Dis.* 72 1634–1638. 10.1136/annrheumdis-2012-202192 23087179

[B75] de Koning H. D. van Gijn M. E. Stoffels M. Jongekrijg J. Zeeuwen P. L. Elferink M. G. (2015). Myeloid lineage-restricted somatic mosaicism of NLRP3 mutations in patients with variant Schnitzler syndrome. *J. Allergy Clin. Immunol.* 135 561–564. 10.1016/j.jaci.2014.07.050 25239704

[B76] De Luca G. Campochiaro C. Dinarello C. A. Dagna L. Cavalli G. (2018a). Treatment of dilated cardiomyopathy with interleukin-1 inhibition. *Ann Intern. Med.* 10.7326/L18-0315 [Epub ahead of print]. 30073251

[B77] De Luca G. Cavalli G. Campochiaro C. Tresoldi M. Dagna L. (2018b). Myocarditis: an interleukin-1-mediated disease? *Front. Immunol.* 9:1335. 10.3389/fimmu.2018.01335 29951067 PMC6008311

[B78] De Luca G. Cavalli G. Baldissera E. Dagna L. (2017). Assessing the role of pentraxin-3 in Takayasu’s arteritis. comment on: plasma pentraxin-3 levels in patients with takayasu’s arteritis during routine follow-up. Alibaz-Oner F. et al. *Clin. Exp. Rheumatol.* 103:221. 28375830

[B79] Delluc A. Limal N. Puechal X. Frances C. Piette J. C. Cacoub P. (2008). Efficacy of anakinra, an IL1 receptor antagonist, in refractory sweet syndrome. *Ann. Rheum. Dis.* 67 278–279. 18192308 10.1136/ard.2006.068254

[B80] DeSena A. D. Do T. Schulert G. S. (2018). Systemic autoinflammation with intractable epilepsy managed with interleukin-1 blockade. *J Neuroinflammation* 15:38. 10.1186/s12974-018-1063-2 29426321 PMC5807745

[B81] Devasahayam J. Pillai U. Lacasse A. (2012). A rare case of pericarditis, complication of infliximab treatment for Crohn’s disease. *J. Crohns Colitis* 6 730–731. 10.1016/j.crohns.2012.02.016 22465046

[B82] Diamond E. L. Abdel-Wahab O. Durham B. H. Dogan A. Ozkaya N. Brody L. (2016). Anakinra as efficacious therapy for two cases of intracranial Erdheim-Chester disease. *Blood* 128 1896–1898. 10.1182/blood-2016-06-725143 27535996 PMC5054701

[B83] Diamond E. L. Dagna L. Hyman D. M. Cavalli G. Janku F. Estrada-Veras J. (2014). Consensus guidelines for the diagnosis and clinical management of Erdheim-Chester disease. *Blood* 124 483–492. 10.1182/blood-2014-03-561381 24850756 PMC4110656

[B84] Dierselhuis M. P. Frenkel J. Wulffraat N. M. Boelens J. J. (2005). Anakinra for flares of pyogenic arthritis in PAPA syndrome. *Rheumatology* 44 406–408. 10.1093/rheumatology/keh479 15637033

[B85] Dinarello C. A. (1996). Biological basis for interleukin-1 in disease. *Blood* 87 2095–2147. 8630372

[B86] Dinarello C. A. (2015). The history of fever, leukocytic pyrogen and interleukin-1. *Temperature* 2 8–16. 10.1080/23328940.2015.1017086 27226996 PMC4843879

[B87] Dinarello C. A. Goldin N. P. Wolff S. M. (1974). Demonstration and characterization of two distinct human leukocytic pyrogens. *J. Exp. Med.* 139 1369–1381. 4829934 10.1084/jem.139.6.1369 PMC2139679

[B88] Dinarello C. A. Ikejima T. Warner S. J. Orencole S. F. Lonnemann G. Cannon J. G. (1987). Interleukin 1 induces interleukin 1. I. Induction of circulating interleukin 1 in rabbits *in vivo* and in human mononuclear cells *in vitro*. *J. Immunol.* 139 1902–1910. 3497982

[B89] Dinarello C. A. Renfer L. Wolff S. M. (1977). Human leukocytic pyrogen: purification and development of a radioimmunoassay. *Proc. Natl. Acad. Sci. U.S.A.* 74 4624–4627. 22079 10.1073/pnas.74.10.4624 PMC431999

[B90] Dinarello C. A. Rosenwasser L. J. Wolff S. M. (1981). Demonstration of a circulating suppressor factor of thymocyte proliferation during endotoxin fever in humans. *J. Immunol.* 127 2517–2519. 6795276

[B91] Dinarello C. A. Simon A. van der Meer J. W. (2012). Treating inflammation by blocking interleukin-1 in a broad spectrum of diseases. *Nat. Rev. Drug Discov.* 11 633–652. 10.1038/nrd3800 22850787 PMC3644509

[B92] Donath M. Y. Shoelson S. E. (2011). Type 2 diabetes as an inflammatory disease. *Nat. Rev. Immunol.* 11 98–107. 10.1038/nri2925 21233852

[B93] Duewell P. Kono H. Rayner K. J. Sirois C. M. Vladimer G. Bauernfeind F. G. (2010). NLRP3 inflammasomes are required for atherogenesis and activated by cholesterol crystals. *Nature* 464 1357–1361. 10.1038/nature08938 20428172 PMC2946640

[B94] Dusser P. Kone-Paut I. (2017). IL-1 inhibition may have an important role in treating refractory Kawasaki disease. *Front. Pharmacol.* 8:163. 10.3389/fphar.2017.00163 28400731 PMC5368266

[B95] Eisenberg S. P. Evans R. J. Arend W. P. Verderber E. Brewer M. T. Hannum C. H. (1990). Primary structure and functional expression from complementary DNA of a human interleukin-1 receptor antagonist. *Nature* 343 341–346. 2137201 10.1038/343341a0

[B96] Eleftheriou D. Gerschman T. Sebire N. Woo P. Pilkington C. A. Brogan P. A. (2011). Biologic therapy in refractory chronic non-bacterial osteomyelitis of childhood. *Rheumatology* 49 1505–1512. 10.1093/rheumatology/keq122 20430869

[B97] Emmenegger U. Reimers A. Frey U. Fux C. Bihl F. Semela D. (2002). Reactive macrophage activation syndrome: a simple screening strategy and its potential in early treatment initiation. *Swiss Med. Wkly.* 132 230–236. 12087489 10.4414/smw.2002.09941

[B98] Emsley H. C. Smith C. J. Georgiou R. F. Vail A. Hopkins S. J. Rothwell N. J. (2005). A randomised phase II study of interleukin-1 receptor antagonist in acute stroke patients. *J. Neurol. Neurosurg. Psychiatry* 76 1366–1372. 16170078 10.1136/jnnp.2004.054882 PMC1739363

[B99] Eungdamrong J. Boyd K. P. Meehan S. A. Latkowski J. A. (2013). Muckle-wells treatment with anakinra. *Dermatol. J.* 19:20720. 24365011

[B100] Everett B. M. Donath M. Y. Pradhan A. D. Thuren T. Pais P. Nicolau J. C. (2018). Anti-inflammatory therapy with canakinumab for the prevention and management of diabetes. *J. Am. Coll. Cardiol.* 71 2392–2401. 10.1016/j.jacc.2018.03.002 29544870

[B101] Ferrero E. Corti A. Haroche J. Belloni D. Colombo B. Berti A. (2016). Plasma chromogranin a as a marker of cardiovascular involvement in Erdheim-Chester disease. *Oncoimmunology* 5:e1181244. 10.1080/2162402X.2016.1181244 27622037 PMC5006912

[B102] Fitzgerald A. A. Leclercq S. A. Yan A. Homik J. E. Dinarello C. A. (2005). Rapid responses to anakinra in patients with refractory adult-onset Still’s disease. *Arthritis Rheum.* 52 1794–1803. 15934079 10.1002/art.21061

[B103] Fleischmann R. M. Schechtman J. Bennett R. Handel M. L. Burmester G. R. Tesser J. (2003). Anakinra, a recombinant human interleukin-1 receptor antagonist (r-metHuIL-1ra), in patients with rheumatoid arthritis: a large, international, multicenter, placebo-controlled trial. *Arthritis Rheum.* 48 927–934. 12687534 10.1002/art.10870

[B104] Fleischmann R. M. Tesser J. Schiff M. H. Schechtman J. Burmester G. R. Bennett R. (2006). Safety of extended treatment with anakinra in patients with rheumatoid arthritis. *Ann. Rheum. Dis.* 65 1006–1012. 16396977 10.1136/ard.2005.048371 PMC1798263

[B105] Franconieri F. Martin-Silva N. de Boysson H. Galateau-Salle F. Emile J. F. Bienvenu B. (2016). Superior efficacy and tolerance of reduced doses of vemurafenib plus anakinra in erdheim-chester disease: towards the paradigm of combined targeting and immune therapies. *Acta Oncol.* 55 930–932. 10.3109/0284186X.2015.1120885 27031008

[B106] Galea J. Ogungbenro K. Hulme S. Greenhalgh A. Aarons L. Scarth S. (2011). Intravenous anakinra can achieve experimentally effective concentrations in the central nervous system within a therapeutic time window: results of a dose-ranging study. *J. Cereb. Blood Flow Metab.* 31 439–447. 10.1038/jcbfm.2010.103 20628399 PMC3049499

[B107] Galeotti C. Tran T.-A. Franchi-Abella S. Fabre M. Pariente D. Kone-Paut I. (2008). IL-1RA agonist (anakinra) in the treatment of multifocal Castleman disease. *J. Pediatr. Hematol. Oncol.* 30 920–924. 10.1097/MPH.0b013e31818ab31f 19131781

[B108] Gamez-Gonzalez L. B. Moribe-Quintero I. Cisneros-Castolo M. Varela-Ortiz J. Munoz-Ramirez M. Garrido-Garcia M. (2018). Kawasaki disease shock syndrome; a unique and severe subtype of kawasaki disease. *Pediatr. Int.* 60 781–790. 10.1111/ped.13614 29888440

[B109] Gatti S. Beck J. Fantuzzi G. Bartfai T. Dinarello C. A. (2002). Effect of interleukin-18 on mouse core body temperature. *Am. J. Physiol. Regul. Integr. Comp. Physiol.* 282 R702–R709. 11832389 10.1152/ajpregu.00393.2001

[B110] Gattorno M. Pelagatti M. A. Meini A. Obici L. Barcellona R. Federici S. (2008a). Persistent efficacy of anakinra in patients with tumor necrosis factor receptor-associated periodic syndrome. *Arthritis Rheum.* 58 1516–1520. 10.1002/art.23475 18438813

[B111] Gattorno M. Piccini A. Lasiglie D. Tassi S. Brisca G. Carta S. (2008b). The pattern of response to anti-interleukin-1 treatment distinguishes two subsets of patients with systemic-onset juvenile idiopathic arthritis. *Arthritis Rheum.* 58 1505–1515. 10.1002/art.23437 18438814

[B112] Gattorno M. Tassi S. Carta S. Delfino L. Ferlito F. Pelagatti M. A. (2007). Pattern of interleukin-1beta secretion in response to lipopolysaccharide and ATP before and after interleukin-1 blockade in patients with CIAS1 mutations. *Arthritis Rheum.* 56 3138–3148. 17763411 10.1002/art.22842

[B113] Genant H. K. Bresnihan B. Ng E. Robbins S. Newmark R. D. McCabe D. (2001). Treatment with anakinra reduces the rate of joint destruction and shows accelerated benefit in the secon 6 months of treatment for patients with rheumatoid arthritis. *Ann. Rheum. Dis.* 40(Suppl. 1):169.

[B114] Gerard S. le Goff B. Maugars Y. Berthelot J. M. Malard O. (2007). Lasting remission of a Muckle-Wells syndrome with CIAS-1 mutation using half-dose anakinra. *Joint Bone Spine* 74:659. 10.1016/j.jbspin.2007.01.032 17892965

[B115] Gerfaud-Valentin M. Jamilloux Y. Iwaz J. Seve P. (2014). Adult-onset still’s disease. *Autoimmun. Rev.* 13 708–722. 10.1016/j.autrev.2014.01.058 24657513

[B116] Goldbach-Mansky R. (2011). Current status of understanding the pathogenesis and management of patients with NOMID/CINCA. *Curr. Rheumatol. Rep.* 13 123–131. 10.1007/s11926-011-0165-y 21538043 PMC3195512

[B117] Goldbach-Mansky R. Dailey N. J. Canna S. W. Gelabert A. Jones J. Rubin B. I. (2006). Neonatal-onset multisystem inflammatory disease responsive to interleukin-1beta inhibition. *N. Engl. J. Med.* 355 581–592. 16899778 10.1056/NEJMoa055137 PMC4178954

[B118] Granowitz E. V. Santos A. Poutsiaka D. D. Cannon J. G. Wilmore D. A. Wolff S. M. (1991). Circulating interleukin-1 receptor antagonist levels during experimental endotoxemia in humans. *Lancet* 338 1423–1424. 1683422 10.1016/0140-6736(91)92725-h

[B119] Grayson P. C. Yazici Y. Merideth M. Sen H. N. Davis M. Novakovich E. (2017). Treatment of mucocutaneous manifestations in Behcet’s disease with anakinra: a pilot open-label study. *Arthritis Res. Ther.* 19:69. 10.1186/s13075-017-1222-3 28335798 PMC5364674

[B120] Grom A. A. (2003). Macrophage activation syndrome and reactive hemophagocytic lymphohistiocytosis: the same entities? *Curr. Opin. Rheumatol.* 15 587–590. 12960485 10.1097/00002281-200309000-00011

[B121] Grom A. A. Mellins E. D. (2011). Macrophage activation syndrome: advances towards understanding pathogenesis. *Curr. Opin. Rheumatol.* 22 561–566. 10.1097/01.bor.0000381996.69261.71 20517154 PMC4443835

[B122] Grom A. A. Villanueva J. Lee S. Goldmuntz E. A. Passo M. H. Filipovich A. (2003). Natural killer cell dysfunction in patients with systemic-onset juvenile rheumatoid arthritis and macrophage activation syndrome. *J. Pediatr.* 142 292–296. 12640378 10.1067/mpd.2003.110

[B123] Guarda G. Braun M. Staehli F. Tardivel A. Mattmann C. Forster I. (2011). Type I interferon inhibits interleukin-1 production and inflammasome activation. *Immunity* 34 213–223. 10.1016/j.immuni.2011.02.006 21349431

[B124] Gul A. Tugal-Tutkun I. Dinarello C. A. Reznikov L. Esen B. A. Mirza A. (2012). Interleukin-1beta-regulating antibody XOMA 052 (gevokizumab) in the treatment of acute exacerbations of resistant uveitis of Behcet’s disease: an open-label pilot study. *Ann. Rheum. Dis.* 71 563–566. 10.1136/annrheumdis-2011-155143 22084392

[B125] Hannum C. H. Wilcox C. J. Arend W. P. Joslin F. G. Dripps D. J. Heimdal P. L. (1990). Interleukin-1 receptor antagonist activity of a human interleukin-1 inhibitor. *Nature* 343 336–340. 10.1038/343336a0 2137200

[B126] Harrison S. R. McGonagle D. Nizam S. Jarrett S. van der Hilst J. McDermott M. F. (2016). Anakinra as a diagnostic challenge and treatment option for systemic autoinflammatory disorders of undefined etiology. *JCI Insight* 1:e86336. 10.1172/jci.insight.86336 27699261 PMC5033915

[B127] Haspolat S. Baysal Y. Duman O. Coskun M. Tosun O. Yegin O. (2005). Interleukin-1alpha, interleukin-1beta, and interleukin-1Ra polymorphisms in febrile seizures. *J. Child Neurol.* 20 565–568. 10.1177/08830738050200070401 16159520

[B128] Hawkins P. N. Lachmann H. J. McDermott M. F. (2003). Interleukin-1 receptor antagonist in the Muckle-Wells syndrome. *N. Engl. J. Med.* 348 2583–2584. 12815153 10.1056/NEJM200306193482523

[B129] Hayashi M. Jin Y. Yorgov D. Santorico S. A. Hagman J. Ferrara T. M. (2016). Autoimmune vitiligo is associated with gain-of-function by a transcriptional regulator that elevates expression of *HLA-A^∗^02:01* in vivo. *Proc. Natl. Acad. Sci. U.S.A.* 113 1357–1362. 10.1073/pnas.1525001113 26787886 PMC4747738

[B130] Heida J. G. Moshe S. L. Pittman Q. J. (2009). The role of interleukin-1beta in febrile seizures. *Brain Dev.* 31 388–393. 10.1016/j.braindev.2008.11.013 19217733 PMC2699664

[B131] Hedrich C. M. Fiebig B. Sallmann S. Bruck N. Hahn G. Roesler J. (2008). Good response to IL-1β blockade by anakinra in a 23-year-old CINCA/NOMID patient without mutations in the *CIAS1* gene. Cytokine profiles and functional studies. *Scand. J. Rheumatol.* 37 385–389. 10.1080/03009740801978889 18609262

[B132] Helmy A. Guilfoyle M. R. Carpenter K. L. Pickard J. D. Menon D. K. Hutchinson P. J. (2014). Recombinant human interleukin-1 receptor antagonist in severe traumatic brain injury: a phase II randomized control trial. *J. Cereb. Blood Flow Metab.* 34 845–851. 10.1038/jcbfm.2014.23 24569690 PMC4013762

[B133] Hennig S. Bayegan K. Uffmann M. Thalhammer F. Winkler S. (2010). Pneumonia in a patient with familial mediterranean fever successfully treated with anakinra-case report and review. *Rheumatol. Int.* 32 1801–1804. 10.1007/s00296-010-1429-y 20352226

[B134] Herlin T. Fiirgaard B. Bjerre M. Kerndrup G. Hasle H. Bing X. (2013). Efficacy of anti-IL-1 treatment in Majeed syndrome. *Ann. Rheum. Dis.* 72 410–413. 10.1136/annrheumdis-2012-201818 23087183 PMC3660147

[B135] Hernandez-Rodriguez J. Segarra M. Vilardell C. Sanchez M. Garcia-Martinez A. Esteban M. J. (2004). Tissue production of pro-inflammatory cytokines (IL-1beta, TNFalpha and IL-6) correlates with the intensity of the systemic inflammatory response and with corticosteroid requirements in giant-cell arteritis. *Rheumatology* 43 294–301. 10.1093/rheumatology/keh058 14679293

[B136] Hirsch L. J. Gaspard N. van Baalen A. Nabbout R. Demeret S. Loddenkemper T. (2018). Proposed consensus definitions for new-onset refractory status epilepticus (NORSE), febrile infection-related epilepsy syndrome (FIRES), and related conditions. *Epilepsia* 59 739–744. 10.1111/epi.14016 29399791

[B137] Hoffman H. M. Wanderer A. A. (2011). Inflammasome and IL-1beta-mediated disorders. *Curr. Allergy Asthma Rep.* 10 229–235. 10.1007/s11882-010-0109-z PMC2892083 20425006

[B138] Honda K. Ohga S. Takada H. Nomura A. Ohshima K. Kinukawa N. (2000). Neuron-specific enolase in hemophagocytic lymphohistiocytosis: a potential indicator for macrophage activation? *Int. J. Hematol.* 72 55–60. 10979210

[B139] Hornung V. Bauernfeind F. Halle A. Samstad E. O. Kono H. Rock K. L. (2008). Silica crystals and aluminum salts activate the NALP3 inflammasome through phagosomal destabilization. *Nat. Immunol.* 9 847–856. 10.1038/ni.1631 18604214 PMC2834784

[B140] Hsiao J. L. Antaya R. J. Berger T. Maurer T. Shinkai K. Leslie K. S. (2011). Hidradenitis suppurativa and concomitant pyoderma gangrenosum: a case series and literature review. *Arch. Dermatol.* 146 1265–1270. 10.1001/archdermatol.2010.328 21079064

[B141] Hsieh C. W. Chen Y. M. Lin C. C. Tang K. T. Chen H. H. Hung W. T. (2017). Elevated expression of the NLRP3 inflammasome and its correlation with disease activity in adult-onset still disease. *J. Rheumatol.* 44 1142–1150. 10.3899/jrheum.161354 28507179

[B142] Ikonomidis I. Lekakis J. P. Nikolaou M. Paraskevaidis I. Andreadou I. Kaplanoglou T. (2008). Inhibition of interleukin-1 by anakinra improves vascular and left ventricular function in patients with rheumatoid arthritis. *Circulation* 117 2662–2669. 10.1161/CIRCULATIONAHA.107.731877 18474811

[B143] Ilowite N. Porras O. Reiff A. Rudge S. Punaro M. Martin A. (2009). Anakinra in the treatment of polyarticular-course juvenile rheumatoid arthritis: safety and preliminary efficacy results of a randomized multicenter study. *Clin. Rheumatol.* 28 129–137. 10.1007/s10067-008-0995-9 18766426

[B144] Imazio M. (2014). Idiopathic recurrent pericarditis as an immune-mediated disease: current insights into pathogenesis and emerging treatment options. *Expert Rev. Clin. Immunol.* 10 1487–1492. 10.1586/1744666X.2014.965150 25307995

[B145] Iurlo A. Dagna L. Cattaneo D. Orofino N. Bianchi P. Cavalli G. (2016). Erdheim-chester disease with multiorgan involvement, following polycythemia vera: a case report. *Medicine* 95:e3697. 10.1097/MD.0000000000003697 27196481 PMC4902423

[B146] Janka G. E. (2012). Familial and acquired hemophagocytic lymphohistiocytosis. *Annu. Rev. Med.* 63 233–246. 10.1146/annurev-med-041610-134208 22248322

[B147] Jeru I. (2011). Role of interleukin-1 in NLRP12-associated autoinflammatory disorders and resistance to anti–. (Interleukin)-1 therapy. *Arthritis Rheum.* 63 2142–2148. 21480187 10.1002/art.30378

[B148] Joosten L. A. Netea M. G. Fantuzzi G. Koenders M. I. Helsen M. M. Sparrer H. (2009). Inflammatory arthritis in caspase 1 gene-deficient mice: contribution of proteinase 3 to caspase 1-independent production of bioactive interleukin-1beta. *Arthritis Rheum.* 60 3651–3662. 10.1002/art.25006 19950280 PMC2993325

[B149] Joosten L. A. Netea M. G. Mylona E. Koenders M. I. Malireddi R. K. Oosting M. (2010). Engagement of fatty acids with Toll-like receptor 2 drives interleukin-1beta production via the ASC/caspase 1 pathway in monosodium urate monohydrate crystal-induced gouty arthritis. *Arthritis Rheum.* 62 3237–3248. 10.1002/art.27667 20662061 PMC2970687

[B150] Junge G. Mason J. Feist E. (2017). Adult onset Still’s disease-The evidence that anti-interleukin-1 treatment is effective and well-tolerated (a comprehensive literature review). *Semin. Arthritis Rheum.* 47 295–302. 10.1016/j.semarthrit.2017.06.006 28757235

[B151] Kamari Y. Werman-Venkert R. Shaish A. Werman A. Harari A. Gonen A. (2007). Differential role and tissue specificity of interleukin-1alpha gene expression in atherogenesis and lipid metabolism. *Atherosclerosis* 195 31–38. 10.1016/j.atherosclerosis.2006.11.026 17173923

[B152] Kanemoto K. Kawasaki J. Miyamoto T. Obayashi H. Nishimura M. (2000). Interleukin (IL)1beta, IL-1alpha, and IL-1 receptor antagonist gene polymorphisms in patients with temporal lobe epilepsy. *Ann. Neurol.* 47 571–574. 10805326

[B153] Kelly A. Ramanan A. V. (2008). A case of macrophage activation syndrome successfully treated with anakinra. *Nat. Clin. Pract. Rheumatol.* 4 615–620. 10.1038/ncprheum0919 18825135

[B154] Kelly C. Hamilton J. (2007). What kills patients with rheumatoid arthritis? *Rheumatology* 46 183–184. 10.1093/rheumatology/kel332 17003170

[B155] Kenney-Jung D. L. Vezzani A. Kahoud R. J. LaFrance-Corey R. G. Ho M. L. Muskardin T. W. (2016). Febrile infection-related epilepsy syndrome treated with anakinra. *Ann. Neurol.* 80 939–945. 10.1002/ana.24806 27770579 PMC5225882

[B156] Killu A. M. Liang J. J. Jaffe A. S. (2013). Erdheim-chester disease with cardiac involvement successfully treated with anakinra. *Int. J. Cardiol.* 167 e115–e117. 10.1016/j.ijcard.2013.04.057 23659884

[B157] Kitley J. L. Lachmann H. J. Pinto A. Ginsberg L. (2010). Neurologic manifestations of the cryopyrin-associated periodic syndrome. *Neurology* 74 1267–1270. 10.1212/WNL.0b013e3181d9ed69 20404307

[B158] Klein A. K. Horneff G. (2011). Improvement of sensoneurinal hearing loss in a patient with muckle-wells syndrome treated with anakinra. *Klin. Padiatr.* 222 266–268. 10.1055/s-0029-1239527 20135584

[B159] Kluger N. Gil-Bistes D. Guillot B. Bessis D. (2011). Efficacy of anti-interleukin-1 receptor antagonist anakinra (Kineret(R)) in a case of refractory Sweet’s syndrome. *Dermatology* 222 123–127. 10.1159/000326112 21464561

[B160] Kone-Paut I. Cimaz R. Herberg J. Bates O. Carbasse A. Saulnier J. P. (2018). The use of interleukin 1 receptor antagonist (anakinra) in Kawasaki disease: a retrospective cases series. *Autoimmun. Rev.* 17 768–774. 10.1016/j.autrev.2018.01.024 29885546

[B161] Kuemmerle-Deschner J. B. Hachulla E. Cartwright R. Hawkins P. N. Tran T. A. Bader-Meunier B. (2011a). Two-year results from an open-label, multicentre, phase III study evaluating the safety and efficacy of canakinumab in patients with cryopyrin-associated periodic syndrome across different severity phenotypes. *Ann. Rheum. Dis.* 70 2095–2102. 10.1136/ard.2011.152728 21859692

[B162] Kuemmerle-Deschner J. B. Lohse P. Koetter I. Dannecker G. E. Reess F. Ummenhofer K. (2011b). NLRP3 E311K mutation in a large family with Muckle-Wells syndrome - description of a heterogeneous phenotype and response to treatment. *Arthritis Res. Ther.* 13:R196. 10.1186/ar3526 22146561 PMC3334646

[B163] Kuemmerle-Deschner J. B. Tyrrell P. N. Koetter I. Wittkowski H. Bialkowski A. Tzaribachev N. (2011c). Efficacy and safety of anakinra therapy in pediatric and adult patients with the autoinflammatory Muckle-Wells syndrome. *Arthritis Rheum.* 63 840–849. 10.1002/art.30149 21360513

[B164] Kuemmerle-Deschner J. B. Wittkowski H. Tyrrell P. N. Koetter I. Lohse P. Ummenhofer K. (2013). Treatment of Muckle-wells syndrome: analysis of two IL-1-blocking regimens. *Arthritis Res. Ther.* 15:R64. 10.1186/ar4237 23718630 PMC4060562

[B165] Kullenberg T. Lofqvist M. Leinonen M. Goldbach-Mansky R. Olivecrona H. (2016). Long-term safety profile of anakinra in patients with severe cryopyrin-associated periodic syndromes. *Rheumatology* 55 1499–1506. 10.1093/rheumatology/kew208 27143789 PMC4957676

[B166] Kumar N. Goyal J. Goel A. Shakoory B. Chatham W. (2014). Macrophage activation syndrome secondary to human monocytic ehrlichiosis. *Indian J. Hematol. Blood Transfus.* 30(Suppl. 1) 145–147. 10.1007/s12288-013-0299-3 25332563 PMC4192266

[B167] Lachmann H. J. Kone-Paut I. Kuemmerle-Deschner J. B. Leslie K. S. Hachulla E. Quartier P. (2009). Use of canakinumab in the cryopyrin-associated periodic syndrome. *N. Engl. J. Med.* 360 2416–2425. 10.1056/NEJMoa0810787 19494217

[B168] Larroche C. Mouthon L. (2004). Pathogenesis of hemophagocytic syndrome (HPS). *Autoimmun. Rev.* 3 69–75. 15003190 10.1016/S1568-9972(03)00091-0

[B169] Larsen C. M. Faulenbach M. Vaag A. Ehses J. A. Donath M. Y. Mandrup-Poulsen T. (2009). Sustained effects of interleukin-1 receptor antagonist treatment in type 2 diabetes. *Diabetes Care* 32 1663–1668. 10.2337/dc09-0533 19542207 PMC2732140

[B170] Larsen C. M. Faulenbach M. Vaag A. Volund A. Ehses J. A. Seifert B. (2007). Interleukin-1-receptor antagonist in type 2 diabetes mellitus. *N. Engl. J. Med.* 356 1517–1526. 17429083 10.1056/NEJMoa065213

[B171] Lee J. K. Kim S. H. Lewis E. C. Azam T. Reznikov L. L. Dinarello C. A. (2004). Differences in signaling pathways by IL-1beta and IL-18. *Proc. Natl. Acad. Sci. U.S.A.* 101 8815–8820. 15161979 10.1073/pnas.0402800101 PMC423278

[B172] Lepore L. Paloni G. Caorsi R. Alessio M. Rigante D. Ruperto N. (2011). Follow-up and quality of life of patients with cryopyrin-associated periodic syndromes treated with Anakinra. *J. Pediatr.* 157 310 e1–315.e1. 10.1016/j.jpeds.2010.02.040 20472245

[B173] Leung C. C. Yu I. T. Chen W. (2012). Silicosis. *Lancet* 379 2008–2018. 10.1016/S0140-6736(12)60235-9 22534002

[B174] Libby P. Ridker P. M. Maseri A. (2002). Inflammation and atherosclerosis. *Circulation* 105 1135–1143. 11877368 10.1161/hc0902.104353

[B175] Lipsker D. Perrigouard C. Foubert A. Cribier B. (2010). Anakinra for difficult-to-treat neutrophilic panniculitis: IL-1 blockade as a promising treatment option for neutrophil-mediated inflammatory skin disease. *Dermatology* 220 264–267. 10.1159/000280436 20197651

[B176] Lomedico P. T. Gubler R. Hellmann C. P. Dukovich M. Giri J. G. Pan Y. E. (1984). Cloning and expression of murine interleukin-1 cDNA in *Escherichia coli*. *Nature* 312 458–462. 6209582 10.1038/312458a0

[B177] Lopalco G. Rigante D. Giannini M. Galeazzi M. Lapadula G. Iannone F. (2016a). Safety profile of anakinra in the management of rheumatologic, metabolic and autoinflammatory disorders. *Clin. Exp. Rheumatol.* 34 531–538. 26940286

[B178] Lopalco G. Vitale A. Iannone F. Cantarini L. (2016b). Anakinra long-term efficacy and safety in the management of Schnitzler’s syndrome and latent tuberculosis infection. *Clin. Exp. Rheumatol.* 34:353. 26940429

[B179] Lopetuso L. R. Chowdhry S. Pizarro T. T. (2013). Opposing functions of classic and novel il-1 family members in gut health and disease. *Front. Immunol.* 4:181. 10.3389/fimmu.2013.00181 23847622 PMC3705591

[B180] Ly K. H. Stirnemann J. Liozon E. Michel M. Fain O. Fauchais A. L. (2013). Interleukin-1 blockade in refractory giant cell arteritis. *Joint Bone Spine* 81 76–78. 10.1016/j.jbspin.2013.06.004 23890680

[B181] Maedler K. Sergeev P. Ris F. Oberholzer J. Joller-Jemelka H. I. Spinas G. A. (2002). Glucose-induced beta cell production of IL-1beta contributes to glucotoxicity in human pancreatic islets. *J. Clin. Invest.* 110 851–860. 12235117 10.1172/JCI15318 PMC151125

[B182] Maeno N. Takei S. Imanaka H. Yamamoto K. Kuriwaki K. Kawano Y. (2004). Increased interleukin-18 expression in bone marrow of a patient with systemic juvenile idiopathic arthritis and unrecognized macrophage-activation syndrome. *Arthritis Rheum.* 50 1935–1938. 15188370 10.1002/art.20268

[B183] Marchi N. Fan Q. Ghosh C. Fazio V. Bertolini F. Betto G. (2009). Antagonism of peripheral inflammation reduces the severity of status epilepticus. *Neurobiol. Dis.* 33 171–181. 10.1016/j.nbd.2008.10.002 19010416 PMC3045783

[B184] Martinon F. Mayor A. Tschopp J. (2009). The inflammasomes: guardians of the body. *Annu. Rev. Immunol.* 27 229–265. 10.1146/annurev.immunol.021908.132715 19302040

[B185] Marzano A. V. Ishak R. S. Saibeni S. Crosti C. Meroni P. L. Cugno M. (2013). Autoinflammatory skin disorders in inflammatory bowel diseases, pyoderma gangrenosum and sweet’s syndrome: a comprehensive review and disease classification criteria. *Clin. Rev. Allergy Immunol.* 45 202–210. 10.1007/s12016-012-8351-x 23334898

[B186] Masters S. L. Dunne A. Subramanian S. L. Hull R. L. Tannahill G. M. Sharp F. A. (2010). Activation of the NLRP3 inflammasome by islet amyloid polypeptide provides a mechanism for enhanced IL-1beta in type 2 diabetes. *Nat. Immunol.* 11 897–904. 10.1038/ni.1935 20835230 PMC3103663

[B187] Mazodier K. Marin V. Novick D. Farnarier C. Robitail S. Schleinitz N. (2005). Severe imbalance of IL-18/IL-18BP in patients with secondary hemophagocytic syndrome. *Blood* 106 3483–3489. 16020503 10.1182/blood-2005-05-1980 PMC1895045

[B188] McDermott M. F. Aksentijevich I. Galon J. McDermott E. M. Ogunkolade B. W. Centola M. (1999). Germline mutations in the extracellular domains of the 55 kDa TNF receptor, TNFR1, define a family of dominantly inherited autoinflammatory syndromes. *Cell* 97 133–144. 10199409 10.1016/s0092-8674(00)80721-7

[B189] McGonagle D. Tan A. L. Madden J. Emery P. McDermott M. F. (2008). Successful treatment of resistant pseudogout with anakinra. *Arthritis Rheum.* 58 631–633. 10.1002/art.23119 18240249

[B190] McGonagle D. Tan A. L. Shankaranarayana S. Madden J. Emery P. McDermott M. F. (2007). Management of treatment resistant inflammation of acute on chronic tophaceous gout with anakinra. *Ann. Rheum. Dis.* 66 1683–1684. 17998217 10.1136/ard.2007.073759 PMC2095323

[B191] Meinzer U. Quartier P. Alexandra J. F. Hentgen V. Retornaz F. Kone-Paut I. (2011). Interleukin-1 targeting drugs in familial mediterranean fever: a case series and a review of the literature. *Semin. Arthritis Rheum.* 41 265–271. 10.1016/j.semarthrit.2010.11.003 21277619

[B192] Mertens M. Singh J. A. (2009a). Anakinra for rheumatoid arthritis. *Cochrane Database Syst. Rev.* 21:CD005121. 10.1002/14651858.CD005121.pub3 19160248 PMC12296252

[B193] Mertens M. Singh J. A. (2009b). Anakinra for rheumatoid arthritis: a systematic review. *J. Rheumatol.* 36 1118–1125. 19447938 10.3899/jrheum.090074

[B194] Miettunen P. M. Narendran A. Jayanthan A. Behrens E. M. Cron R. Q. (2012). Successful treatment of severe paediatric rheumatic disease-associated macrophage activation syndrome with interleukin-1 inhibition following conventional immunosuppressive therapy: case series with 12 patients. *Rheumatology* 50 417–419. 20693540 10.1093/rheumatology/keq218

[B195] Moran A. Bundy B. Becker D. J. DiMeglio L. A. Gitelman S. E. Goland R. (2013). Interleukin-1 antagonism in type 1 diabetes of recent onset: two multicentre, randomised, double-blind, placebo-controlled trials. *Lancet* 381 1905–1915. 10.1016/S0140-6736(13)60023-9 23562090 PMC3827771

[B196] Morton A. C. Rothman A. M. Greenwood J. P. Gunn J. Chase A. Clarke B. (2015). The effect of interleukin-1 receptor antagonist therapy on markers of inflammation in non-ST elevation acute coronary syndromes: the MRC-ILA Heart Study. *Eur. Heart J.* 36 377–384. 10.1093/eurheartj/ehu272 25079365 PMC4320321

[B197] Moser C. Pohl G. Haslinger I. Knapp S. Rowczenio D. Russel T. (2009). Successful treatment of familial Mediterranean fever with anakinra and outcome after renal transplantation. *Nephrol. Dial. Transplant.* 24 676–678. 10.1093/ndt/gfn646 19033248

[B198] Movva R. Brown S. B. Morris D. L. Figueredo V. M. (2013). Anakinra for myocarditis in juvenile idiopathic arthritis. *Tex. Heart Inst. J.* 40 623–625. 24391342 PMC3853823

[B199] Mulders-Manders C. M. Baas M. C. Molenaar F. M. Simon A. (2017). Peri- and postoperative treatment with the interleukin-1 receptor antagonist anakinra is safe in patients undergoing renal transplantation: case series and review of the literature. *Front. Pharmacol.* 8:342. 10.3389/fphar.2017.00342 28620307 PMC5449651

[B200] Murphy P. A. Cebula T. A. Levin J. Windle B. E. (1981). Rabbit macrophages secrete two biochemically and immunologically distinct endogenous pyrogens. *Infect. Immun.* 34 177–183. 7298180 10.1128/iai.34.1.177-183.1981 PMC350840

[B201] Nakayama J. Arinami T. (2006). Molecular genetics of febrile seizures. *Epilepsy Res.* 70(Suppl. 1) S190–S198. 10.1016/j.eplepsyres.2005.11.023 16887333

[B202] Naumann L. Feist E. Natusch A. Langen S. Krause A. Buttgereit F. (2010). IL1-receptor antagonist anakinra provides long-lasting efficacy in the treatment of refractory adult-onset Still’s disease. *Ann. Rheum. Dis.* 69 466–467. 20107032 10.1136/ard.2009.108068

[B203] Netea M. G. Balkwill F. Chonchol M. Cominelli F. Donath M. Y. Giamarellos-Bourboulis E. J. (2017). A guiding map for inflammation. *Nat. Immunol.* 18 826–831. 10.1038/ni.3790 28722720 PMC5939996

[B204] Neven B. Marvillet I. Terrada C. Ferster A. Boddaert N. Couloignier V. (2011). Long-term efficacy of the interleukin-1 receptor antagonist anakinra in ten patients with neonatal-onset multisystem inflammatory disease/chronic infantile neurologic, cutaneous, articular syndrome. *Arthritis Rheum.* 62 258–267. 10.1002/art.25057 20039428

[B205] Nold M. F. Nold-Petry C. A. Zepp J. A. Palmer B. E. Bufler P. Dinarello C. A. (2010). IL-37 is a fundamental inhibitor of innate immunity. *Nat. Immunol.* 11 1014–1022. 10.1038/ni.1944 20935647 PMC3537119

[B206] Norheim K. B. Harboe E. Goransson L. G. Omdal R. (2012). Interleukin-1 inhibition and fatigue in primary Sjogren’s syndrome - a double blind, randomised clinical trial. *PLoS One* 7:e30123. 10.1371/journal.pone.0030123 22253903 PMC3254637

[B207] Novick D. Schwartsburd B. Pinkus R. Suissa D. Belzer I. Sthoeger Z. (2001). A novel IL-18BP ELISA shows elevated serum il-18BP in sepsis and extensive decrease of free IL-18. *Cytokine* 14 334–342. 11497494 10.1006/cyto.2001.0914

[B208] Ogilvie A. C. Hack C. E. Wagstaff J. van Mierlo G. J. Erenberg A. J. Thomsen L. L. (1996). IL-1 beta does not cause neutrophil degranulation but does lead to IL- 6, IL-8, and nitrite/nitrate release when used in patients with cancer. *J. Immunol.* 156 389–394. 8598489

[B209] Ogilvie E. M. Khan A. Hubank M. Kellam P. Woo P. (2007). Specific gene expression profiles in systemic juvenile idiopathic arthritis. *Arthritis Rheum.* 56 1954–1965. 10.1002/art.22644 17530721

[B210] Ogungbenro K. Hulme S. Rothwell N. Hopkins S. Tyrrell P. Galea J. (2016). Study design and population pharmacokinetic analysis of a phase II dose-ranging study of interleukin-1 receptor antagonist. *J. Pharmacokinet. Pharmacodyn.* 43 1–12. 10.1007/s10928-015-9450-0 26476629

[B211] Ozen S. Bilginer Y. Ayaz N. A. Calguneri M. (2011). Anti-interleukin 1 treatment for patients with familial Mediterranean fever resistant to colchicine. *J. Rheumatol.* 38 516–518. 10.3899/jrheum.100718 21159830

[B212] Pacini G. Cavalli G. Tomelleri A. De Luca G. Pacini G. Ferrarini M. (2018). The fibrogenic chemokine CCL18 is associated with disease severity in Erdheim-Chester disease. *Oncoimmunology* 7:e1440929. 10.1080/2162402X.2018.1440929 29900045 PMC5993512

[B213] Pascual V. Allantaz F. Arce E. Punaro M. Banchereau J. (2005). Role of interleukin-1 (IL-1) in the pathogenesis of systemic onset juvenile idiopathic arthritis and clinical response to IL-1 blockade. *J. Exp. Med.* 201 1479–1486. 15851489 10.1084/jem.20050473 PMC2213182

[B214] Pathak S. Goldofsky E. Vivas E. X. Bonagura V. R. Vambutas A. (2011). IL-1beta is overexpressed and aberrantly regulated in corticosteroid nonresponders with autoimmune inner ear disease. *J. Immunol.* 186 1870–1879. 10.4049/jimmunol.1002275 21199898 PMC3031454

[B215] Pazyar N. Feily A. Yaghoobi R. (2012). An overview of interleukin-1 receptor antagonist, anakinra, in the treatment of cutaneous diseases. *Curr. Clin. Pharmacol.* 7 271–275. 22794157 10.2174/157488412803305821

[B216] Peiro C. Lorenzo O. Carraro R. Sanchez-Ferrer C. F. (2017). IL-1beta inhibition in cardiovascular complications associated to diabetes mellitus. *Front. Pharmacol.* 8:363 10.3389/fphar.2017.00363 PMC5468794 28659798

[B217] Picco P. Brisca G. Traverso F. Loy A. Gattorno M. Martini A. (2009). Successful treatment of idiopathic recurrent pericarditis in children with interleukin-1beta receptor antagonist (anakinra): an unrecognized autoinflammatory disease? *Arthritis Rheum.* 60 264–268. 10.1002/art.24174 19116906

[B218] Pomerantz B. J. Reznikov L. L. Harken A. H. Dinarello C. A. (2001). Inhibition of caspase 1 reduces human myocardial ischemic dysfunction via inhibition of IL-18 and IL-1beta. *Proc. Natl. Acad. Sci. U.S.A.* 98 2871–2876. 11226333 10.1073/pnas.041611398 PMC30232

[B219] Prieur A. M. Kaufmann M. T. Griscelli C. Dayer J. M. (1987). Specific interleukin-1 inhibitor in serum and urine of children with systemic juvenile chronic arthritis. *Lancet* 2 1240–1242. 2890857 10.1016/s0140-6736(87)91854-x

[B220] Primdahl J. Clausen J. Horslev-Petersen K. (2013). Results from systematic screening for cardiovascular risk in outpatients with rheumatoid arthritis in accordance with the EULAR recommendations. *Ann. Rheum. Dis.* 72 1771–1776. 10.1136/annrheumdis-2013-203682 23852694

[B221] Punzi L. Gava A. Galozzi P. Sfriso P. (2011). Miscellaneous non-inflammatory musculoskeletal conditions blau syndrome. *Best Pract. Res. Clin. Rheumatol.* 25 703–714. 10.1016/j.berh.2011.10.017 22142748

[B222] Quartier P. Allantaz F. Cimaz R. Pillet P. Messiaen C. Bardin C. (2011). A multicentre, randomised, double-blind, placebo-controlled trial with the interleukin-1 receptor antagonist anakinra in patients with systemic-onset juvenile idiopathic arthritis (ANAJIS trial). *Ann. Rheum. Dis.* 70 747–754. 10.1136/ard.2010.134254 21173013 PMC3070271

[B223] Raffeiner B. Botsios C. Dinarello C. A. Ometto F. Punzi L. Ramonda R. (2011). Adult-onset still’s disease with myocarditis successfully treated with the interleukin-1 receptor antagonist anakinra. *Joint Bone Spine* 78 100–101. 21036649 10.1016/j.jbspin.2010.09.014

[B224] RajasekaranS.KruseK.KoveyK.DavisA. T.HassanN. E.NdikaA. N. (2014). Therapeutic role of anakinra, an interleukin-1 receptor antagonist, in the management of secondary hemophagocytic lymphohistiocytosis/sepsis/multiple organ dysfunction/macrophage activating syndrome in critically ill children^∗^. *Pediatr. Crit. Care Med.* 15 401–408. 10.1097/PCC.0000000000000078 24583503

[B225] Reddy S. Jia S. Geoffrey R. Lorier R. Suchi M. Broeckel U. (2009). An autoinflammatory disease due to homozygous deletion of the IL1RN locus. *N. Engl. J. Med.* 360 2438–2444. 10.1056/NEJMoa0809568 19494219 PMC2803085

[B226] Ridker P. M. Everett B. M. Thuren T. MacFadyen J. G. Chang W. H. Ballantyne C. (2017). Antiinflammatory therapy with canakinumab for atherosclerotic disease. *N. Engl. J. Med.* 377 1119–1131. 10.1056/NEJMoa1707914 28845751

[B227] Ridker P. M. MacFadyen J. G. Everett B. M. Libby P. Thuren T. Glynn R. J. (2018). Relationship of c-reactive protein reduction to cardiovascular event reduction following treatment with canakinumab: a secondary analysis from the CANTOS randomised controlled trial. *Lancet* 391 319–328. 10.1016/S0140-6736(17)32814-3 29146124

[B228] Rigante D. Ansuini V. Caldarelli M. Bertoni B. La Torraca I. Stabile A. (2006). Hydrocephalus in CINCA syndrome treated with anakinra. *Childs Nerv. Syst.* 22 334–337. 10.1007/s00381-006-1280-3 16525848

[B229] Rissanen A. Howard C. P. Botha J. Thuren T. (2012). Effect of anti-IL-1beta antibody (canakinumab) on insulin secretion rates in impaired glucose tolerance or type 2 diabetes: results of a randomized, placebo-controlled trial. *Diabetes Obes. Metab.* 14 1088–1096. 10.1111/j.1463-1326.2012.01637.x 22726220

[B230] Robertson M. J. Mier J. W. Logan T. Atkins M. Koon H. Koch K. M. (2006). Clinical and biological effects of recombinant human interleukin-18 administered by intravenous infusion to patients with advanced cancer. *Clin. Cancer Res.* 12(14 Pt 1) 4265–4273. 10.1158/1078-0432.CCR-06-0121 16857801

[B231] Rosenwasser L. J. Dinarello C. A. Rosenthal A. S. (1979). Adherent cell function in murine T-lymphocyte antigen recognition. IV. Enhancement of murine T-cell antigen recognition by human leukocytic pyrogen. *J. Exp. Med.* 150 709–714. 314491 10.1084/jem.150.3.709 PMC2185642

[B232] Ruiz Gomez A. Couce M. L. Garcia-Villoria J. Torres A. Bana Souto A. Yague J. (2012). Clinical, genetic, and therapeutic diversity in 2 patients with severe mevalonate kinase deficiency. *Pediatrics* 129 e535–e539. 10.1542/peds.2010-2192 22271696

[B233] Ruperto N. Quartier P. Wulffraat N. Woo P. Ravelli A. Mouy R. (2012). A phase II, multicenter, open-label study evaluating dosing and preliminary safety and efficacy of canakinumab in systemic juvenile idiopathic arthritis with active systemic features. *Arthritis Rheum.* 64 557–567. 10.1002/art.33342 21953497

[B234] Ryan J. G. de Koning H. D. Beck L. A. Booty M. G. Kastner D. L. Simon A. (2008). IL-1 blockade in Schnitzler syndrome: ex vivo findings correlate with clinical remission. *J. Allergy Clin. Immunol.* 121 260–262. 17936890 10.1016/j.jaci.2007.09.021

[B235] Rynne M. Maclean C. Bybee A. McDermott M. F. Emery P. (2006). Hearing improvement in a patient with variant Muckle-Wells syndrome in response to interleukin 1 receptor antagonism. *Ann. Rheum. Dis.* 65 533–534. 16531551 10.1136/ard.2005.038091 PMC1798106

[B236] Sakran W. Shalev S. A. Sakran W. Shalev S. A. El-Shanti H. Uziel Y. (2013). Chronic recurrent multifocal osteomyelitis and deficiency of interleukin-1-receptor antagonist. *Pediatr. Infect. Dis. J.* 32:94. 10.1097/INF.0b013e3182700cc1 23241992

[B237] Schellevis M. A. Stoffels M. Hoppenreijs E. P. Bodar E. Simon A. van der Meer J. W. (2011). Variable expression and treatment of PAPA syndrome. *Ann. Rheum. Dis.* 70 1168–1170. 21325428 10.1136/ard.2009.126185

[B238] Schulert G. S. Grom A. A. (2015). Pathogenesis of macrophage activation syndrome and potential for cytokine- directed therapies. *Annu. Rev. Med.* 66 145–159. 10.1146/annurev-med-061813-012806 25386930 PMC5846123

[B239] Scott I. C. Vijay Hajela V. Hawkins P. N. Lachmann H. J. (2011). A case series and systematic literature review of anakinra and immunosuppression in idiopathic recurrent pericarditis. *J. Cardiol. Cases* 4 e93–e97. 30534275 10.1016/j.jccase.2011.07.003 PMC6265095

[B240] Seckinger P. Lowenthal J. W. Williamson K. Dayer J. M. MacDonald H. R. (1987). A urine inhibitor of interleukin 1 activity that blocks ligand binding. *J. Immunol.* 139 1546–1549. 2957429

[B241] Serdaroglu G. Alpman A. Tosun A. Pehlivan S. Ozkinay F. Tekgul H. (2009). Febrile seizures: interleukin 1beta and interleukin-1 receptor antagonist polymorphisms. *Pediatr. Neurol.* 40 113–116. 10.1016/j.pediatrneurol.2008.10.004 19135625

[B242] Settas L. D. Tsimirikas G. Vosvotekas G. Triantafyllidou E. Nicolaides P. (2007). Reactivation of pulmonary tuberculosis in a patient with rheumatoid arthritis during treatment with IL-1 receptor antagonists (anakinra). *J. Clin. Rheumatol.* 13 219–220. 17762459 10.1097/RHU.0b013e31812e00a1

[B243] Shakoory B. Carcillo J. A. Chatham W. W. Amdur R. L. Zhao H. Dinarello C. A. (2016). Interleukin-1 receptor blockade is associated with reduced mortality in sepsis patients with features of macrophage activation syndrome: reanalysis of a prior phase III trial. *Crit. Care Med.* 44 275–281. 10.1097/CCM.0000000000001402 26584195 PMC5378312

[B244] Shukla N. Risen S. Erklauer J. Lai Y. C. Riviello J. Muscal E. (2018). Anakinra (IL-1 blockade) use in children with suspected fires: a single institution experience. *Neurology* 90:346.

[B245] Sibley C. H. Plass N. Snow J. Wiggs E. A. Brewer C. C. King K. A. (2012). Sustained response and prevention of damage progression in patients with neonatal-onset multisystem inflammatory disease treated with anakinra: a cohort study to determine three- and five-year outcomes. *Arthritis Rheum.* 64 2375–2386. 10.1002/art.34409 22294344 PMC3474541

[B246] Simon A. Bodar E. J. van der Hilst J. C. H. Van der Meer J. W. Fiselier T. J. W. Cuppen M. P. J. M. (2004). Beneficial response to interleukin-1 receptor antagonist in TRAPS. *Am. J. Med.* 117 208–210. 15300976 10.1016/j.amjmed.2004.02.039

[B247] Singh J. A. Christensen R. Wells G. A. Suarez-Almazor M. E. Buchbinder R. Lopez-Olivo M. A. (2010). Biologics for rheumatoid arthritis: an overview of cochrane reviews. *Sao Paulo Med. J.* 128 309–310. 21181074 10.1590/S1516-31802010000500013 PMC10948050

[B248] Singh N. Hopkins S. J. Hulme S. Galea J. P. Hoadley M. Vail A. (2014). The effect of intravenous interleukin-1 receptor antagonist on inflammatory mediators in cerebrospinal fluid after subarachnoid haemorrhage: a phase II randomised controlled trial. *J. Neuroinflammation* 11:1. 10.1186/1742-2094-11-1 24383930 PMC3892121

[B249] Sloan-Lancaster J. Abu-Raddad E. Polzer J. Miller J. W. Scherer J. C. De Gaetano A. (2013). Double-blind, randomized study evaluating the glycemic and anti-inflammatory effects of subcutaneous LY2189102, a neutralizing IL-1beta antibody, in patients with type 2 diabetes. *Diabetes Care* 36 2239–2246. 10.2337/dc12-1835 23514733 PMC3714510

[B250] Solovic I. Sester M. Gomez-Reino J. J. Rieder H. L. Ehlers S. Milburn H. J. (2011). The risk of tuberculosis related to tumour necrosis factor antagonist therapies: a TBNET consensus statement. *Eur. Respir. J.* 36 1185–1206. 10.1183/09031936.00028510 20530046

[B251] Sonmez H. E. Demir S. Bilginer Y. Ozen S. (2018). Anakinra treatment in macrophage activation syndrome: a single center experience and systemic review of literature. *Clin. Rheumatol.* 10.1007/s10067-018-4095-1 [Epub ahead of print]. 29663156

[B252] Sparsa L. Afif N. Goetz J. Sordet C. Chatelus E. Lipsker D. (2012). Jessner-Kanof disease induced by leflunomide: a dermal variant of cutaneous lupus? *Rheumatol. Int.* 31 255–258. 10.1007/s00296-009-1169-z 19823837

[B253] Stankovic Stojanovic K. Delmas Y. Torres P. U. Peltier J. Pelle G. Jeru I. (2012). Dramatic beneficial effect of interleukin-1 inhibitor treatment in patients with familial mediterranean fever complicated with amyloidosis and renal failure. *Nephrol. Dial. Transplant.* 27 1898–1901. 10.1093/ndt/gfr528 21931121

[B254] Stew B. T. Fishpool S. J. Owens D. Quine S. (2013). Muckle-Wells syndrome: a treatable cause of congenital sensorineural hearing loss. *B-ENT* 9 161–163. 23909124

[B255] Stienstra R. Joosten L. A. Koenen T. van Tits B. van Diepen J. A. van den Berg S. A. (2011). The inflammasome-mediated caspase-1 activation controls adipocyte differentiation and insulin sensitivity. *Cell Metab.* 12 593–605. 10.1016/j.cmet.2010.11.011 21109192 PMC3683568

[B256] Stoffels M. Simon A. (2011). Hyper-IgD syndrome or mevalonate kinase deficiency. *Curr. Opin. Rheumatol.* 23 419–423. 10.1097/BOR.0b013e328349c3b1 21760510

[B257] Stojanov S. Lapidus S. Chitkara P. Feder H. Salazar J. C. Fleisher T. A. (2011). Periodic fever, aphthous stomatitis, pharyngitis, and adenitis (PFAPA) is a disorder of innate immunity and Th1 activation responsive to IL-1 blockade. *Proc. Natl. Acad. Sci. U.S.A.* 108 7148–7153. 10.1073/pnas.1103681108 21478439 PMC3084055

[B258] Stuyt R. J. Netea M. G. Verschueren I. Dinarello C. A. Kullberg B. J. van der Meer J. W. (2005). Interleukin-18 does not modulate the acute-phase response. *J. Endotoxin. Res.* 11 85–88. 15949134 10.1179/096805105X35170

[B259] Sumegi J. Barnes M. G. Nestheide S. V. Molleran-Lee S. Villanueva J. Zhang K. (2011). Gene expression profiling of peripheral blood mononuclear cells from children with active hemophagocytic lymphohistiocytosis. *Blood* 117 e151–e160. 10.1182/blood-2010-08-300046 21325597 PMC3087540

[B260] Tan Z. S. Beiser A. S. Vasan R. S. Roubenoff R. Dinarello C. A. Harris T. B. (2007). Inflammatory markers and the risk of Alzheimer disease: the Framingham Study. *Neurology* 68 1902–1908. 10.1212/01.wnl.0000263217.36439.da 17536046

[B261] Tilg H. Mier J. W. Vogel W. Aulitzky W. E. Wiedermann C. J. Vannier E. (1993). Induction of circulating IL-1 receptor antagonist by IFN treatment. *J. Immunol.* 150 4687–4692. 8482853

[B262] Toldo S. Mezzaroma E. O’Brien L. Marchetti C. Seropian I. M. Voelkel N. F. (2014). Interleukin-18 mediates interleukin-1-induced cardiac dysfunction. *Am. J. Physiol. Heart Circ. Physiol.* 306 H1025–H1031. 10.1152/ajpheart.00795.2013 24531812 PMC3962640

[B263] Toldo S. Mezzaroma E. Van Tassell B. W. Farkas D. Marchetti C. Voelkel N. F. (2013). Interleukin-1beta blockade improves cardiac remodelling after myocardial infarction without interrupting the inflammasome in the mouse. *Exp. Physiol.* 98 734–745. 10.1113/expphysiol.2012.069831 23180808 PMC6119592

[B264] Tomelleri A. Cavalli G. De Luca G. Campochiaro C. D’Aliberti T. Tresoldi M. (2018). Treating heart inflammation with interleukin-1 blockade in a case of erdheim-chester disease. *Front. Immunol.* 9:1233. 10.3389/fimmu.2018.01233 29910817 PMC5992291

[B265] Tzanetakou V. Kanni T. Giatrakou S. Katoulis A. Papadavid E. Netea M. G. (2016). Safety and efficacy of anakinra in severe hidradenitis suppurativa: a randomized clinical trial. *JAMA Dermatol.* 152 52–59. 10.1001/jamadermatol.2015.3903 26579854

[B266] Uludag I. F. Bilgin S. Zorlu Y. Tuna G. Kirkali G. (2013). Interleukin-6, interleukin-1 beta and interleukin-1 receptor antagonist levels in epileptic seizures. *Seizure* 22 457–461. 10.1016/j.seizure.2013.03.004 23566695

[B267] Uludag I. F. Duksal T. Tiftikcioglu B. I. Zorlu Y. Ozkaya F. Kirkali G. (2015). IL-1beta, IL-6 and IL1Ra levels in temporal lobe epilepsy. *Seizure* 26 22–25. 10.1016/j.seizure.2015.01.009 25799897

[B268] Vambutas A. Lesser M. Mullooly V. Pathak S. Zahtz G. Rosen L. (2014). Early efficacy trial of anakinra in corticosteroid-resistant autoimmune inner ear disease. *J. Clin. Invest.* 124 4115–4122. 10.1172/JCI76503 25133431 PMC4160092

[B269] van Asseldonk E. J. Stienstra R. Koenen T. B. Joosten L. A. Netea M. G. Tack C. J. (2011). Treatment with Anakinra improves disposition index but not insulin sensitivity in nondiabetic subjects with the metabolic syndrome: a randomized, double-blind, placebo-controlled study. *J. Clin. Endocrinol. Metab.* 96 2119–2126. 10.1210/jc.2010-2992 21508140

[B270] van de Veerdonk F. L. Netea M. G. (2013). New Insights in the immunobiology of IL-1 family members. *Front. Immunol.* 4:167. 10.3389/fimmu.2013.00167 23847614 PMC3703542

[B271] van de Veerdonk F. L. Netea M. G. Dinarello C. A. van der Meer J. W. (2011). Anakinra for the inflammatory complications of chronic granulomatous disease. *Neth. J. Med.* 69:95. 21411850

[B272] van der Ven A. J. Netea M. G. van der Meer J. W. de Mast Q. (2015). Ebola virus disease has features of hemophagocytic lymphohistiocytosis syndrome. *Front. Med.* 2:4. 10.3389/fmed.2015.00004 25699258 PMC4316785

[B273] Van Tassell B. W. Abouzaki N. A. Oddi Erdle C. Carbone S. Trankle C. R. Melchior R. D. (2016). Interleukin-1 blockade in acute decompensated heart failure: a randomized, double-blinded, placebo-controlled pilot study. *J. Cardiovasc. Pharmacol.* 67 544–551. 10.1097/FJC.0000000000000378 26906034 PMC5749643

[B274] Van Tassell B. W. Arena R. A. Toldo S. Mezzaroma E. Azam T. Seropian I. M. (2012). Enhanced interleukin-1 activity contributes to exercise intolerance in patients with systolic heart failure. *PLoS One* 7:e33438. 10.1371/journal.pone.0033438 22438931 PMC3306393

[B275] Van Tassell B. W. Canada J. Carbone S. Trankle C. Buckley L. Oddi Erdle C. (2017). Interleukin-1 blockade in recently decompensated systolic heart failure: results from REDHART. *Circ. Heart Fail.* 10:e004373. 10.1161/CIRCHEARTFAILURE.117.004373 29141858 PMC5699505

[B276] Vastert S. J. de Jager W. Noordman B. J. Holzinger D. Kuis W. Prakken B. J. (2014). Effectiveness of first-line treatment with recombinant interleukin-1 receptor antagonist in steroid-naive patients with new-onset systemic juvenile idiopathic arthritis: results of a prospective cohort study. *Arthritis Rheumatol.* 66 1034–1043. 10.1002/art.38296 24757154

[B277] Vezzani A. Maroso M. Balosso S. Sanchez M. A. Bartfai T. (2011). IL-1 receptor/toll-like receptor signaling in infection, inflammation, stress and neurodegeneration couples hyperexcitability and seizures. *Brain Behav. Immun.* 25 1281–1289. 10.1016/j.bbi.2011.03.018 21473909

[B278] Vitale A. Insalaco A. Sfriso P. Lopalco G. Emmi G. Cattalini M. (2016). A snapshot on the on-label and off-label use of the interleukin-1 inhibitors in italy among rheumatologists and pediatric rheumatologists: a nationwide multi-center retrospective observational study. *Front. Pharmacol.* 7:380. 10.3389/fphar.2016.00380 27822185 PMC5076463

[B279] Wada T. Muraoka M. Yokoyama T. Toma T. Kanegane H. Yachie A. (2013). Cytokine profiles in children with primary epstein-barr virus infection. *Pediatr. Blood Cancer* 60 E46–E48. 10.1002/pbc.24480 23382108

[B280] Waghmare S. Valecka B. Cairns A. P. (2015). A severe case of adult onset Stills isease with myopericarditis, resistant to treatment with tocilizumab but responsive to anakinra. *Ulster Med. J.* 84 130–132. 26376493

[B281] Wendling D. Prati C. Aubin F. (2012). Anakinra treatment of SAPHO syndrome: short-term results of an open study. *Ann. Rheum. Dis.* 71 1098–1100. 22219141 10.1136/annrheumdis-2011-200743

[B282] Zarchi K. Dufour D. N. Jemec G. B. (2013). Successful treatment of severe hidradenitis suppurativa with anakinra. *JAMA Dermatol.* 149 1192–1194. 10.1001/jamadermatol.2013.5377 23966107

[B283] Zufferey P. So A. (2013). A pilot study of IL-1 inhibition in acute calcific periarthritis of the shoulder. *Ann. Rheum. Dis.* 72 465–467. 10.1136/annrheumdis-2012-202380 23144448

