# Corrigendum: Anakinra Therapy for Non-cancer Inflammatory Diseases

**DOI:** 10.3389/fphar.2019.00148

**Published:** 2019-03-08

**Authors:** Giulio Cavalli, Charles A. Dinarello

**Affiliations:** ^1^Unit of Immunology, Rheumatology, Allergy and Rare Diseases, San Raffaele Hospital, Vita-Salute San Raffaele University, Milan, Italy; ^2^Department of Medicine, Radboud University Medical Center, Nijmegen, Netherlands; ^3^Department of Medicine, University of Colorado Denver, Denver, CO, United States

**Keywords:** interleukin 1, IL-1β, IL-1α, rheumatology, inflammation

In the original article “Anakinra Therapy for Non-cancer Inflammatory Diseases”, we failed to provide citations for publications relevant to the discovery of IL-1Ra. The citations have now been inserted in the **Introduction**, **Historical Background of IL-1 and IL-1Ra** and should read:

“As stated in our review (Cavalli and Dinarello, 2018), in 1981 we described a circulating suppressor factor from humans during experimental endotoxemia as assayed for specific inhibition of IL-1 activity *in vitro* (Dinarello et al., 1981). We believe this circulating suppressor factor was the first description of IL-1Ra, and we confirmed our findings in a report published in *Lancet* in 1991 using a specific radioimmunoassay for IL-1Ra (Granowitz et al., 1991). However, in 1984, there was documentation from the group of Jean-Michel Dayer describing a specific inhibitor of IL-1 activity isolated from the urine of patients with monoblastic leukemia (Balavoine et al., 1984). This was an essential contribution to the history of the discovery of the antagonist. In 1985, there was another report from the Dayer laboratory “Collagenase- and PGE2-Stimulating Activity (Interleukin-1-Like) and Inhibitor in Urine from a Patient with Monocytic Leukemia”, as published in *Progress in Leukocyte Biology*, vol. 2 (New York, NY: Alan R. Liss, 1985 p. 429). These reports were followed by another publication in the *Journal of Clinical Investigation* (Balavoine et al., 1986). As stated in our Review, “the IL-1 inhibitor” isolated from the urine was shown to prevent binding of IL-1 to cells (Seckinger et al., 1987), thus providing for the first time evidence for its mechanism of action. Because of the widespread and beneficial use of anakinra (the recombinant form of the nature IL-1Ra) to treat human diseases, the contributions of Jean-Michel Dayer as well as those of William Arend are paramount.”

The authors apologize for this error and state that this does not change the scientific conclusions of the article in any way. The original article has been updated.
